# AtDAT1 Is a Key Enzyme of D-Amino Acid Stimulated Ethylene Production in *Arabidopsis thaliana*

**DOI:** 10.3389/fpls.2019.01609

**Published:** 2019-12-12

**Authors:** Juan Suarez, Claudia Hener, Vivien-Alisa Lehnhardt, Sabine Hummel, Mark Stahl, Üner Kolukisaoglu

**Affiliations:** Center for Plant Molecular Biology (ZMBP), University of Tübingen, Tübingen, Germany

**Keywords:** D-amino acids in plants, D-amino acid-stimulated ethylene production, D-amino acid specific transaminase, D-methionine, 1-aminocyclopropane-1-carboxylic acid, ethylene, amino acid malonylation

## Abstract

D-Enantiomers of proteinogenic amino acids (D-AAs) are found ubiquitously, but the knowledge about their metabolism and functions in plants is scarce. A long forgotten phenomenon in this regard is the D-AA-stimulated ethylene production in plants. As a starting point to investigate this effect, the *Arabidopsis* accession Landsberg *erecta* (L*er*) got into focus as it was found defective in metabolizing D-AAs. Combining genetics and molecular biology of T-DNA insertion lines and natural variants together with biochemical and physiological approaches, we could identify AtDAT1 as a major D-AA transaminase in *Arabidopsis*. *Atdat1* loss-of-function mutants and *Arabidopsis* accessions with defective *AtDAT1* alleles were unable to produce the metabolites of D-Met, D-Ala, D-Glu, and L-Met. This result corroborates the biochemical characterization, which showed highest activity of AtDAT1 using D-Met as a substrate. Germination of seedlings in light and dark led to enhanced growth inhibition of *atdat1* mutants on D-Met. Ethylene measurements revealed an increased D-AA stimulated ethylene production in these mutants. According to initial working models of this phenomenon, D-Met is preferentially malonylated instead of the ethylene precursor 1-aminocyclopropane-1-carboxylic acid (ACC). This decrease of ACC degradation should then lead to the increase of ethylene production. We could observe a reciprocal relation of malonylated methionine and ACC upon D-Met application and significantly more malonyl-methionine in *atdat1* mutants. Unexpectedly, the malonyl-ACC levels did not differ between mutants and wild type. With AtDAT1, the first central enzyme of plant D-AA metabolism was characterized biochemically and physiologically. The specific effects of D-Met on ACC metabolism, ethylene production, and plant development of *dat1* mutants unraveled the impact of AtDAT1 on these processes; however, they are not in full accordance to previous working models. Instead, our results imply the influence of additional factors or processes on D-AA-stimulated ethylene production, which await to be uncovered.

## Introduction

It is widely accepted that proteinogenic L-amino acids (L-AAs) are essential in all kingdoms of life, both as primary metabolites as well as elementary building blocks of proteins. In contrast, the metabolism and functions of the D-forms of amino acids (D-AAs) are far less clear. Major reasons for this discrepancy are the large diversity and different functions of D-AAs in organisms. For instance, bioactive peptides like octopine from octopus and scallop, antibiotics from bacteria, and opioids from frogs were among the first substances reported to contain D-AAs (Fujii, 2002; Martínez-Rodríguez et al., 2010; Ollivaux et al., 2014). In humans, several proteins related to diseases like arteriosclerosis, Alzheimer, or Parkinson contain D-AAs, especially D-Asp that are generated by racemization of the corresponding L-AA (Fujii et al., 2011). Various free D-AAs were detected in different tissues and fluids of humans and other mammals (Hamase et al., 2002; Hamase, 2007). The most prominent example in this respect is the impact of D-Asp and D-Ser on the functions of the N-methyl-D-aspartate (NMDA) receptor in mammals: Aberrant levels of these D-AAs seem to be connected with psychological disorders and diseases of the endocrine system [for reviews, see Fuchs et al. (2005); D’aniello, 2007; Katane and Homma (2011); Balu and Coyle (2015)].

Far less is known about the metabolism and functions of D-AAs in plants. This is astonishing against the background that plant roots are surrounded by D-AAs, mainly D-Ala and D-Glu, as degradation products of the peptidoglycan layer of bacterial cell walls (Dworkin, 2014). Thus, the amount of D-AAs in the rhizosphere can be more than 10% of the corresponding L-enantiomer (Brodowski et al., 2005; Amelung et al., 2006). This led to the question if D-AAs are actively utilized by plants. For a long time, D-AAs were considered as toxins due to the fact that some of them inhibit seedling growth in submillimolar concentrations (Erikson et al., 2004; Forsum et al., 2008). However, several reports suggested that D-AAs take up a similarly crucial position in plants as in microbes and animals [for further readings about D-AAs in microbes and animals, see Konno et al. (2007) and Brückner (2011)]. For instance, the D-Ala amount in duckweed (*Landoltia punctata*) was demonstrated to increase during UV light stress (Monselise et al., 2015). Furthermore, D-Ser is involved in pollen tube growth in *Arabidopsis* by regulating the glutamate receptor GLR1.2, which belongs to a group of plant proteins closely related to mammalian NMDA receptors (Michard et al., 2011; Forde and Roberts, 2014). In mosses (*Physcomitrella patens*), D-Ala and D-Glu were detected in the plastidial envelope, similar to bacterial peptidoglycan (Hirano et al., 2016). This finding and others led to the conclusion that peptidoglycan, containing D-Ala and D-Glu, is an integral part of the plastidial envelope not only in cryptophytes [for a review, see Chen et al. (2018)].

The number of enzymes predicted to be specific for processing D-AAs annotated in plant genomes implies much more functions for these AAs than currently known (Naranjo-Ortíz et al., 2016). However, it also raises the question about their metabolism in plants, especially how the abundance of different D-AAs is regulated. On the one hand, their content has to be maintained at required levels to ensure their activity. On the other hand, the intracellular concentrations must be limited below toxic levels. This restriction is of specific importance due to the facts that the rhizosphere is the major natural source of D-AAs for plants (Vranova et al., 2012) and that D-AAs are taken up by roots in considerable amounts (Hill et al., 2011; Gördes et al., 2013). In this respect, the question arises which processes facilitate the catabolism of D-AAs in plants.

In the course of our previous studies, D-Met got into our focus because of its highest conversion rates in almost all tested accessions of *Arabidopsis thaliana* except in L*er* (Gördes et al., 2013), although methionine represents a relatively small portion of soil amino acids (Vranova et al., 2012). But it had been detected in soil (Amelung and Zhang, 2001), and there have also been several bacterial species isolated from soil that are specialized to the utilization of D-Met as sole carbon and nitrogen source (Radkov et al., 2016). Furthermore, it is produced by different bacteria, incorporated into their cell wall and even released to their environment in order to disassemble biofilms [for a review, see Cava et al. (2011)]. Nevertheless, D-Met has not been reported yet to be produced by plants.

More than 30 years ago, it was reported that feeding D-Met and other D-AAs to seedlings of cocklebur (*Xanthium pennsylvanicum*), pumpkin (*Cucurbita moschata*), sunflower (*Helianthus annuus*), mung bean (*Vigna radiata*), water melon (*Citrullus vulgaris*), and pea (*Pisum sativum*) leads to increased ethylene production (Satoh and Esashi, 1980; Liu et al., 1983; Kionka and Amrhein, 1984). This phenomenon was characterized as “D-amino-acid-stimulated ethylene production” (Satoh and Esashi, 1980). The authors tried to explain the effect by competitive malonylation of D-Met and 1-aminocyclopropane-1-carboxylic acid (ACC), the precursor of ethylene. According to this hypothesis, D-Met would compete with ACC for the same malonyl transferase (Liu et al., 1983; Ling-Yuan et al., 1985; Benichou et al., 1995; Wu et al., 1995), which would lead to an increase of ACC level and subsequently ethylene production (Yang and Hoffman, 1984). However, this hypothesis could not be verified because the corresponding malonyl transferase has not been identified to date.

As shown previously, *Arabidopsis* plants are able to convert particular D-AAs like D-Met, D-Trp, D-Phe, and D-His to their respective L-enantiomers (Gördes et al., 2011). Additionally, the feeding of almost all tested D-AAs led mainly to the formation of D-Ala and D-Glu. In contrast, the *Arabidopsis* accession Landsberg *erecta* (L*er*) is incapable of both the D-AA to L-AA and the D-AA to D-Ala/D-Glu conversion (Gördes et al., 2013). These observations point to a central metabolic step, in which D-AAs, with a high preference to D-Met, are converted to D-Ala and D-Glu by a D-AA specific transaminase (Vranova et al., 2012; Gördes et al., 2013).

Here, we describe the identification and characterization of *Arabidopsis* loss-of-function mutant alleles in the Columbia-0 (Col-0) accession for a previously characterized D-AA specific transaminase D-AAT (Funakoshi et al., 2008), which we named AtDAT1. This enzyme has been shown before to have a second enzymatic function as an aminodeoxychorismate lyase (ADCL) in the synthesis of p-aminobenzoate, a folate precursor (Basset et al., 2004). Nevertheless, a physiological role could not be assigned to the AtDAT1 encoding gene in plants to date. Most interestingly, the homolog of *AtDAT1* in *Plasmodium falciparum* also displays such a dual function and the ADCL activity is repressed by D-AAs (Magnani et al., 2013). Loss-of-function mutants of *AtDAT1* showed almost identical defects as L*er* in D-AA metabolism, with D-Met as strongest effector. Indeed, we could show that the affected gene in L*er* encodes for an almost non-functional AtDAT1 isoform. Biochemical analyses revealed that this enzyme prefers D-Met as amino donor and pyruvate over 2-oxoglutarate as amino acceptor, confirming the preferential production of D-Ala in Col-0. The discovery of *AtDAT1* and its mutants gave us also the opportunity to verify the working model of D-AA-stimulated ethylene production in plants. We found that D-Met application causes significantly higher ethylene production and growth inhibition in *atdat1* seedlings compared to wild type. According to the current working model, the increase in ethylene should be caused by a decrease in malonylation of ACC due to the increase of malonyl-D-Met, leading to a higher ACC oxidation. Although we found higher malonyl-methionine concentrations in *atdat1* seedlings after D-Met application, the malonyl-ACC levels decreased equally in mutants and their respective wild type. This points to an additional, yet unraveled, mechanism regulating D-AA-stimulated ethylene production in plants. Nevertheless, our findings indicate functions of D-Met in defined plant processes beyond unspecific growth inhibition.

## Materials and Methods

### Plant Material and Growth Conditions

All *Arabidopsis* ecotypes as well as T-DNA insertion lines analyzed in this study were either provided by the Nottingham Arabidopsis Stock Centre (University of Nottingham, UK) or the Arabidopsis Biological Resource Center (University of Ohio, Columbus, OH).

Seedlings for amino acid extraction and profiling were germinated in microtiter plates as described before (Gördes et al., 2013). For phenotypic analysis of seedlings and subsequent measurement of malonylated methionine and ACC in their extracts, plants were either germinated for 6 days in darkness or 10 days in light (all at 22 °C). As solid growth media ½ MS basal salts with 1% sucrose and 1% phytoagar, including conditional further additions (e.g., D-AAs, ACC) were applied. For all analyses of adult plants, these were grown in the greenhouse in soil.

### PCR Genotyping and RT-PCR Analysis of *Arabidopsis* Lines and Accessions

Plant DNA for PCR analysis was extracted from seedlings or leaves of adult plants according to Edwards et al. (1991). To determine zygosity of T-DNA insertion lines, either a gene specific primer and a border primer or two gene specific primers flanking the insertion (for primer combinations and sequences see **Table S1**) were used in a PCR reaction with Taq polymerase from New England Biolabs (Frankfurt am Main, Germany) according to manufacturer’s protocol. To determine the *AtDAT1* sequence in different *Arabidopsis* ecotypes, the complete coding sequences were amplified from genomic DNA and cDNA as described above and the PCR products were sequenced directly by GATC (Konstanz, Germany). For cDNA synthesis RNA of 14 days old seedlings germinated in liquid media under long day conditions was extracted with the RNeasy Mini Kit from Qiagen (Düsseldorf, Germany) and cDNA was synthesized with RevertAid H Minus Reverse Transcriptase from Thermo Fisher Scientific (Karlsruhe, Germany), both according to manufacturers’ protocols. This cDNA was used for cloning purposes (see below) and RT-PCR analysis.

### Cloning of *AtDAT1* Variants for Recombinant Expression

For cloning *AtDAT1* from cDNA of *Arabidopsis* accessions Col-0 and L*er*, the complete coding sequence was amplified with KOD DNA Polymerase from Merck Millipore (Schwalbach am Taunus, Germany) with the primer combination DAT1-Start/DAT1-A1 (**Table S1**). PCR products were cloned into pENTR/D-TOPO according to manufacturer’s protocol (Thermo Fisher Scientific, Karlsruhe, Germany), leading to the constructs pENTR-AtDAT1_(Col-0)_ and pENTR-AtDAT1_(L_*_er_*_)_. To create *AtDAT1* coding sequences with the single point mutations A77T and T303S, the previously described clones were cleaved with *Pst I* and *Not I*, creating a 0.5 kb fragment. This was then ligated from pENTR-AtDAT1_(Col-0)_ to pENTR-AtDAT1_(L_*_er_*_)_ and vice versa, resulting in the constructs pENTR-AtDAT1_(A77T)_ and pENTR-AtDAT1_(T303S)_. After sequence verification of the constructs, they were all used for LR reaction using the kit from Invitrogen (Karlsruhe, Germany) according to manufacturer’s protocol into pGEX-2TM-GW (kindly provided by Bekir Ülker) for expression in *E. coli* with N-terminal GST tag and C-terminal His tag. Additionally, the pENTR-AtDAT1_(Col-0)_ and pENTR-AtDAT1_(L_*_er_*_)_ were used for Gateway-based cloning into pUB-DEST-GFP for expression in plants with C-terminal GFP tag. pENTR-AtDAT1_(Col-0)_ was used for Gateway-based cloning into pUB-DEST (Grefen et al., 2010) for complementing *AtDAT1* defective plants.

### *Arabidopsis* Transformation and Tobacco Leaf Infiltration

All plant transformation vectors were transformed into *Agrobacterium tumefaciens* cv. pMP90-RK GV3101. Plant transformation was performed by floral dipping (Clough and Bent, 1998). For selection of transformants, seeds were either germinated on ½ MS-Agar with 1% sucrose containing hygromycin or germinated on soil and sprayed with 2% BASTA from AgrEvo (Düsseldorf, Germany) depending on the used vector.

For tobacco leaf infiltration transformed *Agrobacterium* containing pUB10-GFP::DAT1 was mixed with a strain of transformed *Agrobacterium* for expression of the mCherry plastid marker (CD3-999 pt-rk; Nelson et al., 2007) and P19 *A. tumefaciens* cells into infiltration media [10 mM MES-KOH (pH 5.7), 10 mM, MgCl_2,_ 0.2 mM Acetosyringone]. Using a syringe 1 ml of infiltration media with the mix of the three types of cells was infiltrated in the abaxial side of *Nicotiana benthamiana* leaves. Plants were then watered and kept on the lab bench for 2 days. Afterwards, single leaf discs were excised for confocal fluorescence microscopy.

### Fluorescence Microscopy

Imaging was performed using a Leica laser scanning microscope SP8 with the corresponding software LCS or LASAF X (Leica Microsystems, Wetzlar, Germany). For excitation of GFP-fusion proteins, the Argon laser was used at 488 nm and the detection range was from 500 to 550 nm. For m-RFP excitation was set to 561 nm and detection was from 600 to 650 nm. All autofluorescence of chloroplasts was detected in the range from 670 to 725 nm.

### Promoter::GUS Transgenic Analysis

The promoter region from -677 to +11 of the genomic locus of *AtDAT1* from Col-0 and L*er* were amplified by PCR with the primer pair ProDAT1-SGW/ProDAT1-AGW (for sequences, see **Table S1**). The respective fragment was cloned into pENTR/D-TOPO and then into pMDC163 (Curtis and Grossniklaus, 2003), to be transformed into *Arabidopsis* by *Agrobacterium*-mediated gene transfer.

Histochemical staining of GUS activity was analyzed in plants of the T2-generation that had been germinated on liquid media. For GUS staining seedlings and adult plants were washed in sodium phosphate buffer and afterwards incubated overnight at 37°C in this buffer containing 1 mM X-Gluc (5-bromo-4-chloro-3-indolyl-beta-D-glucuronic acid) and 0.5 mM K_3_Fe(CN)_6_. Afterwards chlorophyll was removed for documentation by several washings with hot ethanol.

### Recombinant Expression of *AtDAT1* Variants in *E. coli*

*E. coli* strain BL21(DE3) RIL was transformed with cDNA of *AtDAT1* variants in pGEX-2TM-GW (see above) and grown in LB medium with appropriate antibiotics until they reached an OD_600_ of 0.5. Then expression was induced by addition to a final concentration of 0.1 mM isopropyl-β-D-galactoside (IPTG) and the culture was grown for 20 h at 18°C. Afterwards cells were pelleted by centrifugation and washed once with TE buffer including 100 mM NaCl. After further centrifugation, cells were resuspended in 20 mM Tris, pH 8, with Protease Inhibitor Cocktail from Biotool (Oberasbach, Germany). This suspension was sonicated and afterwards centrifuged with 18,000 × g to clear the crude extract from cell debris.

The recombinant His-tagged AtDAT1 protein variants from this crude extract were purified with Protino Ni-NTA agarose from Macherey-Nagel, (Weilmünster Germany) according to manufacturer’s protocol. Therefore, the column was equilibrated and loaded with 10 mM imidazole, washed with 20 mM imidazole, and elution of His-tagged proteins was achieved with 250 mM imidazole. Imidazole was removed by dialysis with Float-A-Lyzer Dialysis Device from Roth (Karlsruhe, Germany) in 10 mM potassium phosphate, pH 8. Protein content was determined with the Bio-Rad Protein Assay (Bio-Rad, München, Germany) according to manufacturer’s protocol. Specific detection of His tagged proteins on a western blot was achieved with a monoclonal His Tag antibody conjugated to alkaline phosphatase (antikoerper-online.de, Aachen, Germany).

### Enzyme Assays to Determine D-AA Specific Aminotransferase Activity

The standard reaction mixture with 2-OG as amino group acceptor contained D-Ala (10 mM), 2-OG (50 mM), and pyridoxalphosphate (PLP; 50 µM) in potassium phosphate buffer (100 mM, pH 8). For assays with pyruvate as amino group acceptor, D-Ala and 2-OG were replaced by D-Met (10 mM) and pyruvate (50 mM), respectively. To determine substrate specificity, the tested D-AAs were all applied in 10 mM concentration. All assay reactions in triplicates were started by addition of 3–8 µg of purified protein, incubated at 37°C, and samples were taken at different time points up to 90 min. Each sample was derivatized and the amino acids measured as described below.

For the determination of K_M_ and V_max_ values different D-Met concentrations (0.1, 0.5, 1.0, 2.0, 5.0, 10.0, 20.0, and 50.0 mM D-Met) have been incubated with the enzyme AtDAT1 and pyruvate as cosubstrate (50 mM). Produced D-Alanine was analyzed after 0, 5, and 10 min. With the means of three biological replicates for any D-Met concentration and time point, the slope of the time course was calculated and normalized to the protein amount used. To determine K_M_ and V_max_ values, a linearization according to Hofstee (1959) was used.

### Amino Acid Extraction and Determination From Plant Material

Amino acid extraction and derivatization was performed as described before (Gördes et al., 2011). The incubation time of derivatization was elongated to 3 h and the derivatized liquid volume was adjusted with acetonitrile instead of methanol.

Almost all experiments were focused on the measurement of D/L-Alanine, D/L-Glutamate, and D/L-Methionine. To determine and quantify these amino acids in plant extracts and enzyme assays, standard materials were purchased from Sigma-Aldrich (Steinheim, Germany). Other chemicals were obtained in LC/MS grade from Roth (Karlsruhe, Germany). An Acquity–SynaptG2 UPLC-MS system from Waters (Manchester, England) was used for quantification, operated in positive electrospray ionization mode. The mass spectrometer was operated at a capillary voltage of 3,000 V and a resolution of 20,000. Separation of the amino acids was carried out on a Waters Acquity C_18_ HSS T3, 1.0 × 150 mm, 1.8 µm column with a flow rate of 50 µl/min and a 22 min gradient from 70% water to 99% methanol (both with 0.1 % formic acid). For quantification, 3 µl of sample was injected and a 5-point calibration from 0.125 to 1,250 µM was used.

The quantification of malonyl-methionine ([M+H^+^] 218.022) and malonyl-ACC ([M+H^+^] 188.050) was performed relatively using the same LC/MS system described above. However, the stationary phase was changed into a Waters Acquity C_18_ HSS T3, 2.1 × 100 mm, 1.8 µm column, and a flow rate of 0.2 ml/min with a 15 min gradient from 99% water to 99% methanol (both with 0.1% formic acid) was used for separation. The malonylated compounds were identified by the exact mass of their molecular ion followed by a MS/MS fragmentation.

### Analysis of Ethylene

For assaying ethylene production, *Arabidopsis* seedlings were grown in glass vials (18 ml) containing 3 ml solid medium (30 seedlings per vial) for 6 days. The vials were closed with rubber septa and opened once before measuring. After 30–90 min of further incubation, ethylene accumulating in the free air space was measured by gas chromatography using a gas chromatograph equipped with a flame-ionization detector (Felix et al., 1991).

### Statistical Evaluation

Data were analyzed with IBM SPSS Statistics 24. Significance levels were analyzed using an independent two-sided Student’s t-test. For further analyses between and within genotypes, we used an ANOVA followed by *post hoc* tests, Gabriel, or Games-Howell, depending on the equality of variances. For testing the homogeneity of variances, a Levene test was applied.

## Results

### *AtDAT1* as a Candidate Gene for D-AAs Metabolism

Initially, we observed the strong decrease of both D-AA to L-AA and especially D-AA to D-Ala/D-Glu conversion rates in L*er* in comparison to other ecotypes (Gördes et al., 2013). According to the transamination hypothesis, the mutation of at least one D-AA specific transaminase could be responsible for this metabolic phenotype. One candidate protein had been previously identified biochemically to be such an enzyme, named AtDAAT1 (Funakoshi et al., 2008). To investigate its role *in planta* we started to analyze T-DNA insertion lines of the corresponding gene (At5g57850; afterwards designated as *AtDAT1*) regarding their D-AA metabolism.

**Figure 1:**
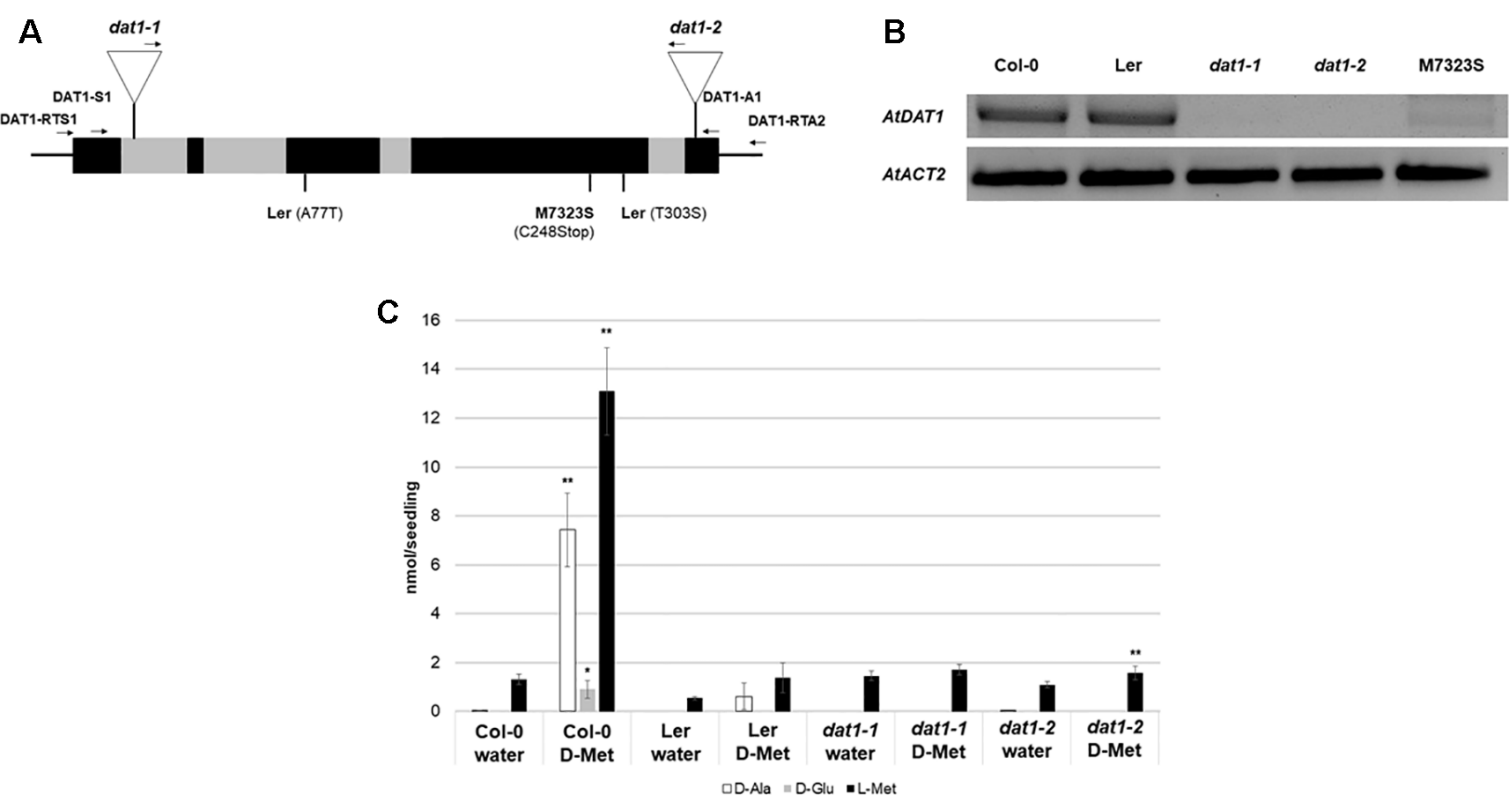
AtDAT1 as a candidate protein for the metabolism of D-AAs in *Arabidopsis*. **(A)** Scheme of the genomic structure of *AtDAT1* (exons and introns in black and grey, respectively) with the positions of T-DNA insertions in *dat1-1* and *dat1-2* as well as the mutations found in L*er* and M7323S. Arrows indicate primers used for genotyping the T-DNA insertions and RT-PCR (for primer sequences, see **Table S1**). **(B)** RT-PCR analysis of *AtDAT1* expression in Col-0, L*er*, *dat1-1, dat1-2*, and M7323S (top: *AtDAT1*; bottom: *AtACT2*). **(C)** Contents of D-Ala (white), D-Glu (gray), and L-Met (black) in seedlings of Col-0, L*er*, *dat1-1*, and *dat1-2* without (water) and with D-Met treatment for 16 h (D-Met). For each measurement four seedlings were pooled and further processed. Error bars represent the standard deviation from three independent measurements. The asterisks indicate the significance level (t-test) of differences of all measurements to the respective line without D-Met treatment (*p < 0.05; **p < 0.01).

Homozygous plants of such insertion lines, SALK_011686 and SALK_111981 (denoted as *dat1-1* and *dat1-2*, respectively; **Figure 1A**), were isolated and propagated for further analyses (see **Table S1** for primer sequences). RT-PCR analysis of *AtDAT1* expression displayed no transcripts with the given primer combination in *dat1-1* and *dat1-2* mutants compared to the corresponding wild type (Col-0) (**Figure 1B**). As observed previously (Lempe et al., 2005), the *AtDAT1* transcript level in L*er* seedlings was similar to that of wild-type Col-0. Feeding with D-Met caused the highest accumulation of D-Ala, D-Glu, and its respective L-enantiomer in Col-0 seedlings of all tested D-AAs. Therefore, seedlings of the *dat1-1* and *dat1-2* mutants, Col-0 and Ler were grown for 14 days on liquid ½ MS medium in light, then supplemented with D-Met and subsequently analyzed for their AA contents. In sharp contrast to Col-0, both *AtDAT1* insertion mutants were neither able to produce D-Ala, D-Glu, nor additional L-Met after application of D-Met. This AA profile was similar to that found in seedlings of the L*er* accession (**Figure 1C**).

Further *in silico* analyses of public transcriptomic data (Lempe et al., 2005) revealed that the accession M7323S displayed a strongly reduced *AtDAT1* transcript level, which could be confirmed by RT-PCR (**Figure 1B**). When this accession was grown on D-Met supplemented medium, defects in AA metabolism were observed (**Figure S1**) similar to those found in L*er* and the *dat1* mutant seedlings. This defect was not just due to the reduced transcription of *AtDAT1* in M7323S. Sequencing of the genomic locus and the cDNA of *AtDAT1* from M7323S revealed that this gene contains a T→A mutation at genomic position +1259. This leads to a nonsense mutation at the third position of a cysteine codon (TGT) to a stop codon (TGA) at position 248 of the AA sequence (C248STOP) (**Figure 1A**). In contrast, sequencing of the genomic locus and the cDNA of *AtDAT1* from L*er* revealed two missense mutations leading to AA exchanges of the protein sequence (A77T and T303S) (**Figure 1A**).

**Figure 2:**
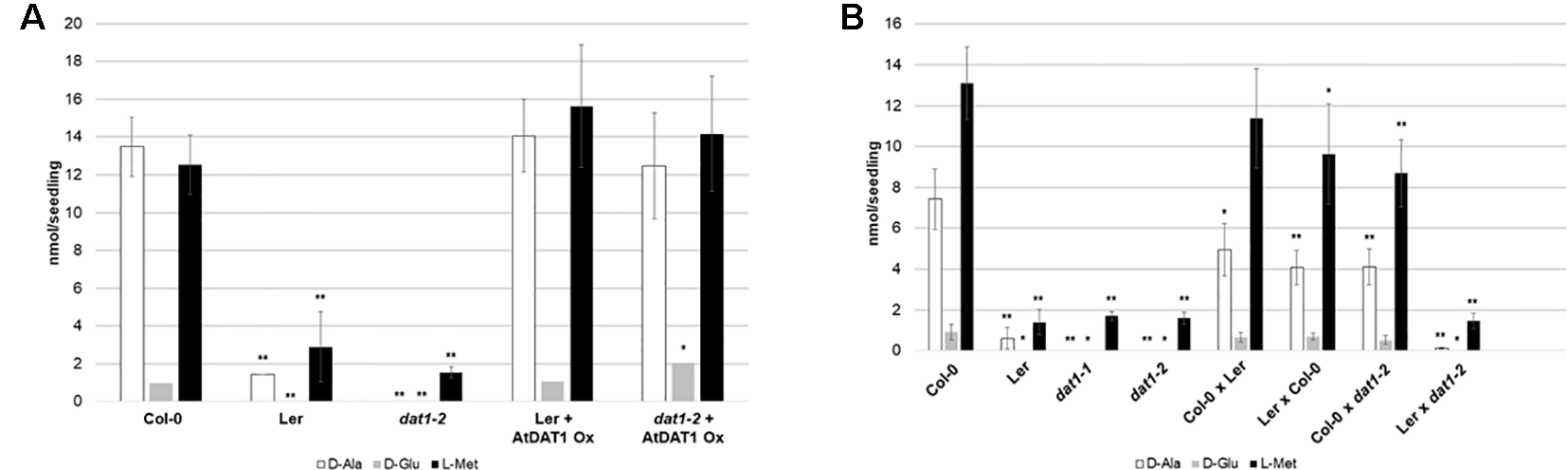
D-Met metabolism in lines overexpressing *AtDAT1* and in F1 seedlings from crosses of Col-0, L*er* and *dat1-2*. Contents of D-Ala, D-Glu, and L-Met after overnight exposure to D-Met **(A)** in L*er* and *dat1-2* seedlings overexpressing *AtDAT1* (AtDAT1 Ox) and their corresponding background lines and **(B)** in seedlings of F1 progeny of crosses of Col-0, L*er*, and *dat1-2* and their corresponding parental lines; for further information, see **Figure 1C** (*p < 0.05; **p < 0.01).

To examine whether these mutations in the *AtDAT1* L*er* allele are responsible for the metabolic aberrations in this accession, we performed different genetic approaches. First, ubiquitin promoter-driven expression of the *AtDAT1* Col-0 allele in transgenic L*er* plants led to the reconstitution of the D-Met metabolism in L*er* and its complementation in the *dat1-2* mutant (**Figure 2A**). Second, F1 seedlings derived from crosses between Col-0 and L*er* and between Col-0 and *dat1-2* displayed no defects in D-Met metabolism as observed in L*er* and *dat1-2*, irrespective of the maternal origin, whereas the offspring of the L*er* x *dat1-2* crossing did (**Figure 2B**). These data prove the defect of *AtDAT1* function in the L*er* accession and the *dat1-2* insertion mutant.

To answer the remaining question about the reason for this defect in L*er*, the expression of *AtDAT1* was analyzed. As mentioned before, the *AtDAT1* transcript levels appeared similar in Col-0 and L*er* (**Figure 1B**). This observation was supported by analysis of transgenic plants containing the *uidA* reporter gene (GUS) under the control of the *AtDAT1* promoter either from the Col-0 or L*er* allele (**Figures S2A**, **B**). There, it can be seen that the reporter constructs are active in seedlings and adult plants and with less GUS staining in late floral stages and seeds (**Figure S2A**), corresponding to expression patterns displayed in the eFP browser (Winter et al., 2007). The activity of the *AtDAT1* promoters derived from Col-0 and L*er* showed no apparent differences, irrespective of the presence of L-Met or D-Met in the media (**Figure S2B**). Subcellular mislocalization would have been another explanation for affected *AtDAT1* function in L*er*. Therefore, GFP-tagged *AtDAT1* gene variants derived from cDNA of both ecotypes expressed under the control of the ubiquitin 10 promoter were transiently transformed into tobacco leaves (**Figure S3**). The Col-0 as well as the Ler cDNA derived AtDAT1 fusion proteins localized to the chloroplasts, as it had been shown before for GFP-tagged AtDAT1_(Col-0)_ (Basset et al., 2004). Therefore, a possible mis-expression of *AtDAT1* or its mis-localization of AtDAT1-GFP in L*er* does not cause the aberrant D-Met metabolism in this accession.

### A Missense Mutation of the *AtDAT1* L*er* Allele Leads to an Almost Complete Loss of the Enzymatic Activity

To clarify if the enzyme encoded by the L*er AtDAT1* allele is able to transaminate D-AAs, the L*er* (AtDAT1_(L_*_er_*_)_) and Col-0 (AtDAT1_(Col-0)_) versions of AtDAT1 were expressed with an N-terminal GST-tag in *E. coli*. After purification by affinity chromatography (for purification results, see **Figure S4**), their enzymatic activities were tested according to Funakoshi et al. (2008).

**Figure 3:**
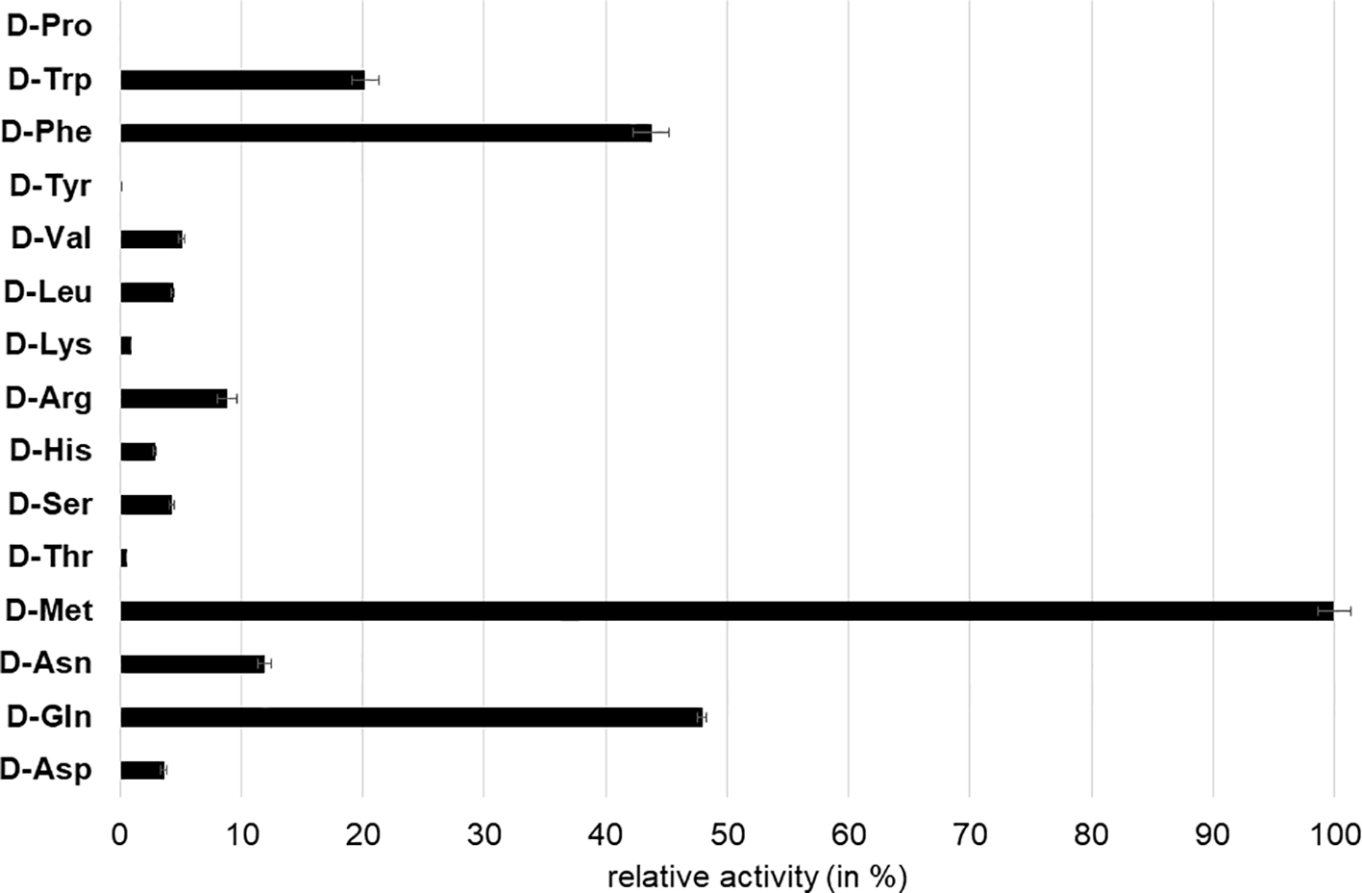
Relative D-Ala producing activity of AtDAT1 with different D-AAs as amino group donor and pyruvate as acceptor. Activity of reaction with D-Met was set to 100% and all other reactions were calculated in relation to it. Each bar represents the mean of measurement of three independent assays. Error bars (± SD).

We first tested AtDAT1_(Col-0)_ for its capability to transaminate 2-oxoglutarate (2-OG) or pyruvate using 16 different D-AAs as amino group donors. With 2-OG used as amino group acceptor, a transaminase reaction was only detectable for the donors D-Met, D-Trp, and D-Ala (**Table S2**), whereas with pyruvate as acceptor, almost all D-AAs, with the exception of D-Pro, led to the formation of D-Ala (**Figure 3**). Furthermore, we measured an over 100 times higher activity for the enzymatic reaction with pyruvate as acceptor than with 2-OG, irrespective of the D-AA applied as amino group donor (**Table S2**). The comparison of the AtDAT1_(Col-0)_ activities using different D-AAs and pyruvate as substrates revealed that D-Met was the best tested amino group donor (**Figure 3**). Using pyruvate and D-Met as substrates, we determined the K_M_ and V_max_ of AtDAT1_(Col-0)_ to be 17.4 mM and 0.07 nkat, respectively.

**Figure 4:**
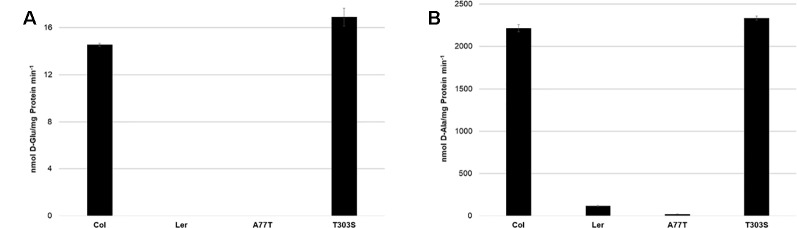
Activities of AtDAT1 variants. Transaminase activities of AtDAT1_(Col-0)_, AtDAT1_(L_*_er_*_)_, AtDAT1_(A77T)_, and AtDAT1_(T303S)_ with D-Met as amino group donor and **(A)** 2-oxoglutarate or **(B)** pyruvate as acceptor molecule are displayed; for further information, see **Figure 3**.

To characterize the activity of AtDAT1_(L_*_er_*_)_ in comparison to AtDAT1_(Col-0)_, enzymatic assays were performed with two substrate combinations: first, with D-Met as amino group donor and pyruvate as acceptor, respectively, as the best substrate combination for AtDAT1_(Col-0)_ and, second, with D-Ala as amino group donor and 2-OG as acceptor. As shown in **Figures 4A** and B for both substrate combinations, the activity of AtDAT1_(L_*_er_*_)_ dropped to 0–5% compared to that of AtDAT1_(Col-0)_.

We next addressed the question whether only one of the missense mutations in AtDAT1_(L_*_er_*_)_ (A77T or T303S) is sufficient to cause the activity loss. The alignment of DAT1 amino acid sequences from different plant species revealed that the alanine at position 77 is more conserved than the threonine at position 303 (**Figure S5**). To analyze the impact of the mutations, AtDAT1_(Col-0)_ derived isoforms harboring single amino acid exchanges of AtDAT1_(Ler)_ were also expressed as N-terminal GST fusions in *E. coli*. The recombinant proteins were affinity-purified and tested for their activity. The enzyme isoform with the T303S amino acid exchange AtDAT1_(T303S)_ showed an activity comparable to AtDAT1_(Col-0)_ (**Figures 4A**, **B**). In contrast, the mutation A77T led to a strong decrease in the production of D-Glu (**Figure 4A**) and D-Ala (**Figure 4B**) with 2-oxoglutarate or pyruvate as substrates, respectively. Instead, the enzymatic defect of AtDAT1_(A77T)_ was quantitatively similar to that of AtDAT1_(L_*_er_*_)_. From these data, we conclude that solely the A77T amino acid exchange is responsible for the activity loss of AtDAT1_(L_*_er_*_)_. Furthermore, the enzymatic data also revealed that the L*er* variant of AtDAT1 is not completely inactive with about 5% remaining activity in comparison to Col-0 (**Figure 4B**).

### The Loss of *AtDAT1* Leads to Decreased Seedling Growth in Response to D-Met

**Figure 5:**
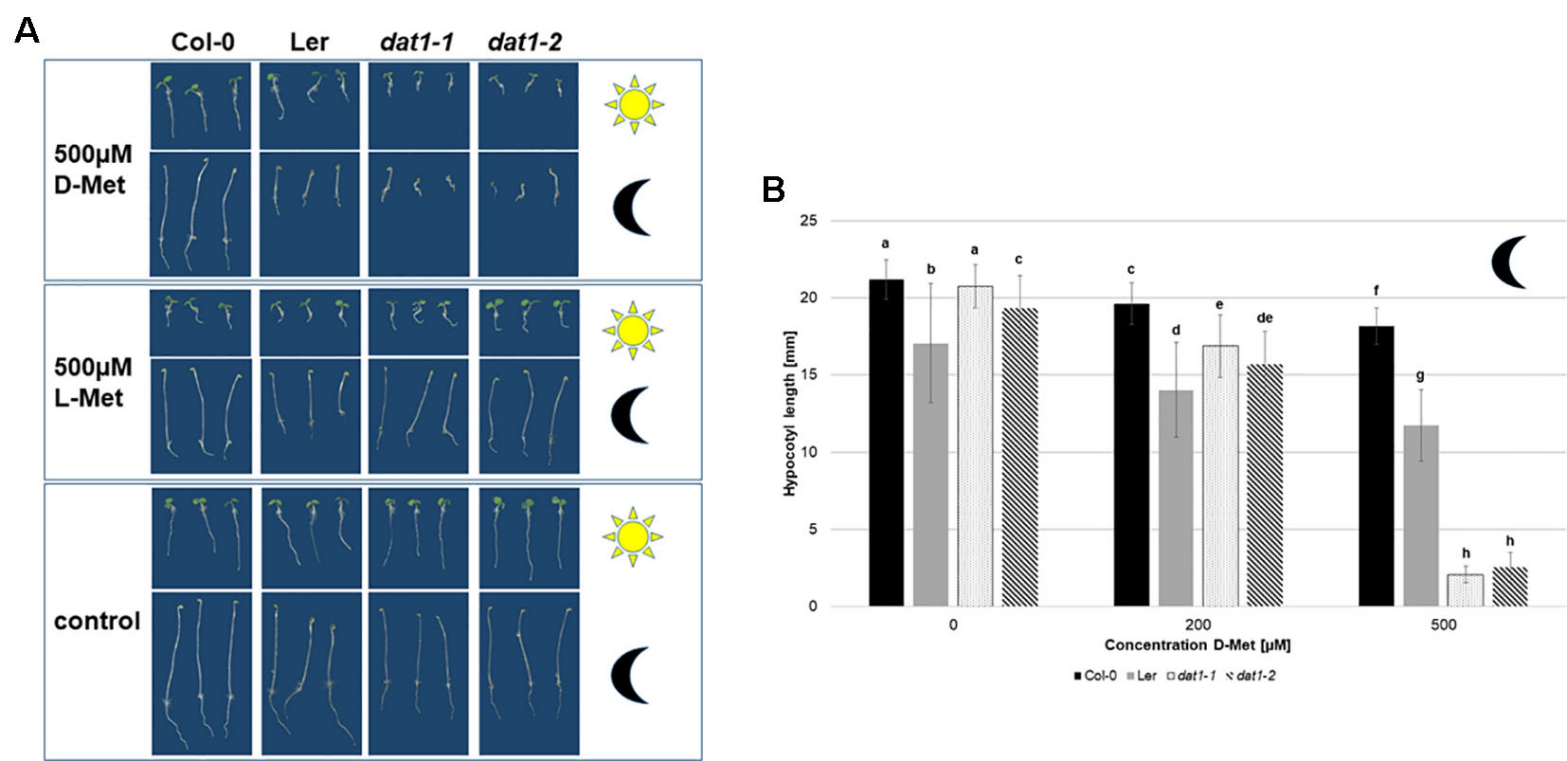
Seedling growth is differentially suppressed by D-Met in *AtDAT1* knock out-lines. **(A)** Seeds of Col-0, *dat1-1*, and *dat1-2*, and L*er* were germinated either in continuous light (sun) or darkness (moon) on different solid growth media (with 500 µM D-Met, with 500 µM L-Met supplemented or without supplementation). **(B)** Hypocotyl growth of the before mentioned dark grown plants. The bars (Col-0: black, L*er*: grey, *dat1-1*: dotted, *dat1-2*: striped; n = 30) represent the average hypocotyl length. Different letters indicate statistically significant differences (p < 0.05) tested by an ANOVA. Error bars (± SD).

After identification of AtDAT1 as a central enzyme of D-AA metabolism, the question arose whether the loss of *AtDAT1* gene function leads to defects in *Arabidopsis* growth and development. Under greenhouse conditions in soil growth of *dat1-1* and *dat1-2* mutant plants could not be distinguished from Col-0 (**Figure S6**). We next asked of how the mutant lines and L*er* would grow in presence of D-Met. Growth of *dat1-1* and *dat1-2* seedlings on media containing 500 µM D-Met resulted in a retardation compared to the corresponding wild type, whereas L*er* took an intermediate response (**Figure 5A**). Testing this growth behavior on the dark-grown etiolated seedlings revealed an even more pronounced growth difference between the *dat1* mutants and Col-0 (**Figure 5B**). All these growth differences were specific for D-Met, whereas the addition of the same concentrations of L-Met did not lead to these differential effects (**Figure 5A**). Altogether, D-Met inhibited seedling growth specifically in *AtDAT1* affected lines.

The reduced growth of hypocotyls and roots of *dat1-1* and *dat1-2* seedlings, especially in the dark (**Figure 5A**), reminded of phenotypes caused by the gaseous plant hormone ethylene. This gets even clearer with a look on the hypocotyl length of the four dark grown lines (**Figure 5B**): There was a highly significant decrease of *dat1-1* and *dat1-2* hypocotyl length of about one-eighth compared to Col-0 grown on 500 µM D-Met. Although increasing L-Met concentrations also led to shorter hypocotyls, this effect was similar in mutant and wild-type plants. Furthermore, the growth inhibition was by far not as strong as with D-Met (**Figure S7**).

### *AtDAT1* Mutants Display Enhanced D-AA Stimulated Ethylene Production

**Figure 6:**
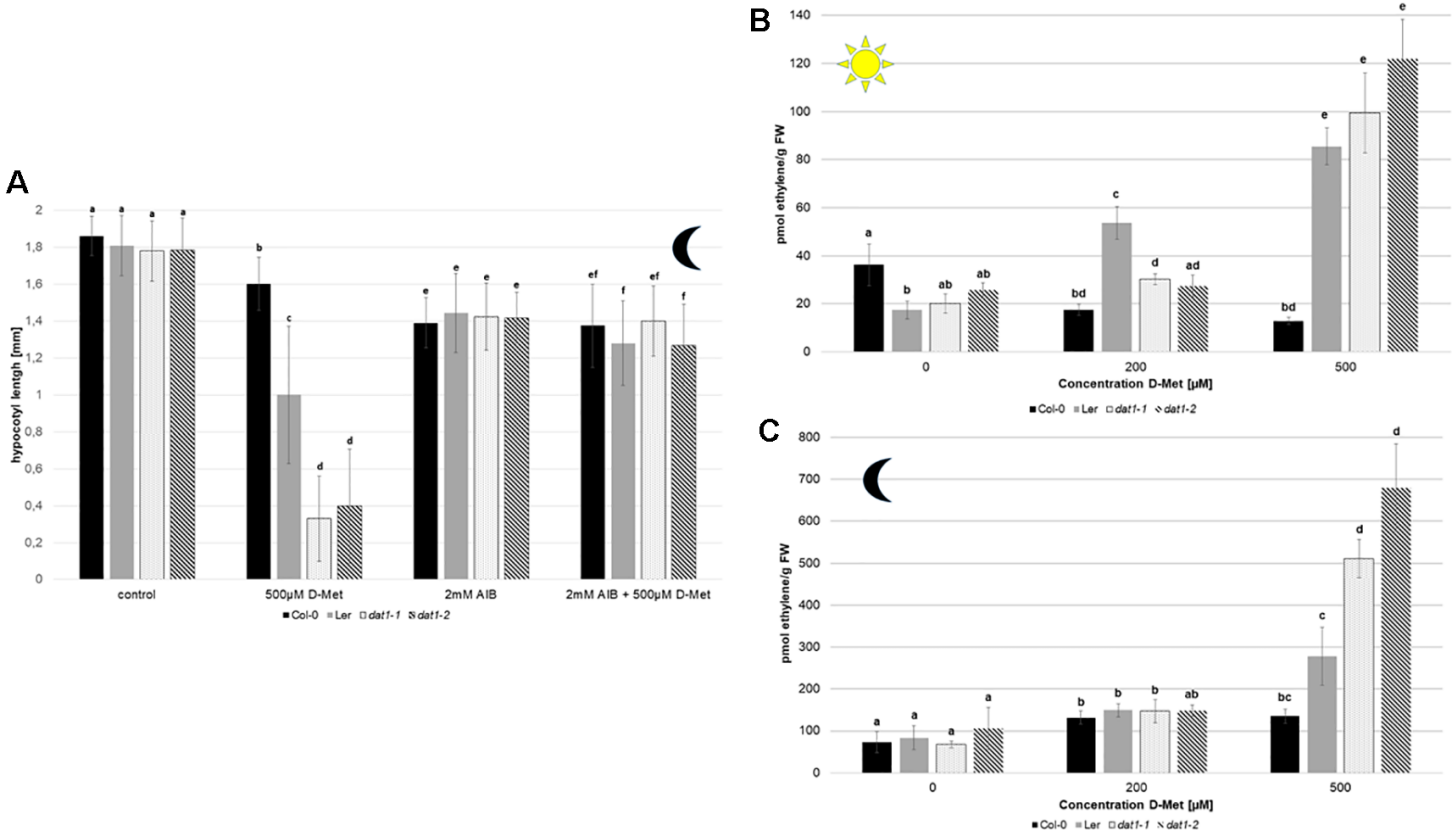
D-Met leads to an increase of ethylene in *AtDAT1* knock out-lines. **(A)** Seeds of Col-0, *dat1-1*, and *dat1-2*, and L*er* were germinated in continuous darkness on solid growth media without any supplementation (control), supplemented just with 500 µM D-Met, supplemented just with 2 mM AIB, and supplemented with both agents together. For further information, see **Figure 5B**. **(B)** Ethylene contents in seedlings of Col-0, L*er*, *dat1-1*, and *dat1-2* were measured after growth in continuous light or **(C)** in darkness in vials with solid growth media supplemented with 200 and 500 µM D-Met, and additionally without supplementation. The bars (Col-0: black, L*er*: gray, *dat1-1*: dotted, *dat1-2*: striped) represent the average values of three biological replicates. Different letters indicate statistically significant differences (p < 0.05) tested by an ANOVA. Error bars (± SD).

To test whether ethylene synthesis is indeed affected in *dat1* mutants by D-Met, we added α-aminoisobutyric acid (AIB) to the growth medium, which leads to the inhibition of ACC oxidase, the enzyme catalyzing the last step of ethylene synthesis (Satoh and Esashi, 1980). As shown in **Figure 6A**, the addition of 2 mM AIB to the growth medium led to a reversion of growth reduction by D-Met of all *dat1* affected lines in the dark. This indicates that the increased ethylene production in these lines is caused by D-Met in the medium.

To elucidate if ethylene production was indeed altered, we measured its content in L*er*, the *dat1* mutants, and Col-0 grown in continuous light and dark. The addition of 500 µM D-Met was sufficient to induce a significant increase of up to threefold of ethylene production in light grown L*er* and *dat1* mutants compared to Col-0 (**Figure 6B**). Even stronger changes in ethylene production could be observed for both *dat1* mutant seedlings grown in the presence of D-Met in the dark, whereas L*er* displayed again an intermediate phenotype (**Figure 6C**).

**Figure 7:**
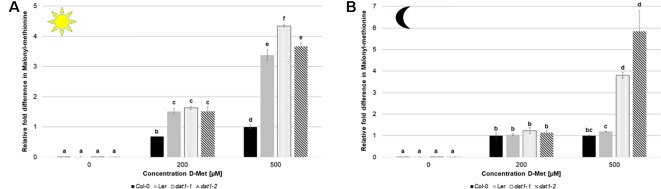
D-Met affects formation of malonyl-methionine differently in Col-0, L*er*, and *dat1* mutants. Malonyl-methionine contents in seedlings of Col-0, L*er*, *dat1-1*, and *dat1-2* were measured after growth **(A)** in continuous light or **(B)** in darkness on agar plates supplemented with 200 and 500 µM D-Met, and additionally without supplementation. The relative values are given in fold changes with the values of Col-0 at 500 µM D-Met set to 1. The bars (Col-0: black, L*er*: gray, *dat1-1*: dotted, *dat1-2*: striped) represent the average values of three biological replicates. Different letters indicate statistically significant differences (p < 0.05) tested by an ANOVA. Error bars (± SD).

As mentioned above, the increase of ethylene production by D-AAs was attributed to competitive malonylation of D-AAs instead of ACC, which should lead to enhanced ACC oxidation resulting in higher ethylene concentration (Yang and Hoffman, 1984). To verify this assumption, we measured the contents of malonyl-methionine and malonyl-ACC in D-Met treated seedlings. In these measurements, we detected a significant increase of malonyl-methionine in Col-0, L*er*, and *dat1* seedlings upon D-Met treatment (**Figures 7A**, **B**). This accumulation was far higher (up to fivefold) in the *dat1* mutants compared to the corresponding wild type, irrespective of the light regime (**Figures 7A**, **B**). Furthermore, L*er* also showed a D-Met induced over-accumulation of malonyl-methionine in the light (**Figure 7A**), but not in darkness (**Figure 7B**).

**Figure 8:**
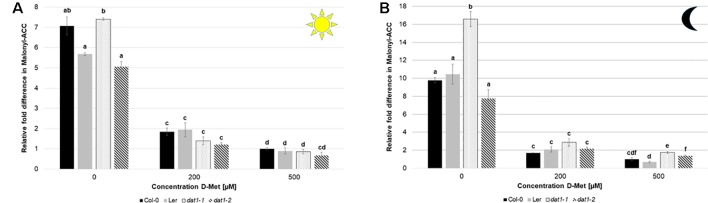
D-Met leads to a decrease of malonyl-ACC levels in all tested lines. Malonyl-ACC contents in seedlings of Col-0, L*er*, *dat1-1*, and *dat1-2* were measured after growth **(A)** in continuous light or **(B)** in darkness on agar plates supplemented with 200 and 500 µM D-Met, and without supplementation. Additionally, all plates contained 10 µM ACC. The relative values are given in fold changes with the values of Col-0 at 500 µM D-Met + 10 µM ACC set to 1. The bars (Col-0: black, L*er*: gray, *dat1-1*: dotted, *dat1-2*: striped) represent the average values of three biological replicates. Different letters indicate statistically significant differences (p < 0.05) tested by an ANOVA. Error bars (± SD).

Since the amount of malonyl-ACC in these experiments was below our detection limit, we added 10 µM ACC to the media and measured the malonyl-ACC in the seedlings. In this case, we were able to detect large amounts of malonyl-ACC in the seedlings of all genotypes, which decreased drastically upon D-Met addition (**Figures 8A**, **B**). It must be noted that the treatment of seedlings with 200 or 500 µM D-Met together with 10 µM ACC is relatively extreme and probably does not reflect physiological conditions. However, D-Met induced malonyl-ACC reduction was undue to production of malonyl-methionine caused by ACC, which was comparable with and without ACC addition (**Table S3**). Nevertheless, there was no significant difference of malonyl-ACC reduction of L*er* and *dat1* mutants to Col-0 at higher D-Met concentrations (**Figures 8A**, **B**).

## Discussion

For several decades, the detrimental, but partially also beneficial, effects of D-AAs on plants have been investigated (Valdovinos and Muir, 1965; Aldag and Young, 1970; Erikson et al., 2004; Erikson et al., 2005; Gördes et al., 2011; Hill et al., 2011). It is noteworthy, that there are reports of some D-AAs synthesized *de novo* by plants (Brückner and Westhauser, 2003; Strauch et al., 2015). However, there is growing evidence in recent years that almost all D-enantiomers of proteinogenic L-AAs are taken up by plants (Aldag and Young, 1970; Forsum et al., 2008; Gördes et al., 2011; Hill et al., 2011) and also metabolized to significant amounts (Aldag and Young, 1970; Gördes et al., 2011). With the proof provided in the actual report, the long standing question was addressed how D-AAs are utilized in plants.

In the light of the observations of Gördes et al. (2011), three possible mechanisms for the metabolism of D-AAs in plants had been suggested: racemization, deamination, and transamination of D-AAs (Vranova et al., 2012; Gördes et al., 2013). Our data indicate that transamination by AtDAT1 is responsible for major steps of D-AA turnover in *Arabidopsis*. This is reflected by its broad range of D-AAs transaminated, although its turnover rate and its affinity is low. Furthermore, we showed that the major product of this enzymatic reaction is D-Ala with D-Met as the favored amino group donor. D-Ala was also preferentially produced when plants were fed with other D-AAs. The preferred synthesis of D-Ala is caused by the preference of AtDAT1 on pyruvate over 2-OG as substrate. In comparison to the work of Funakoshi et al. (2008), who used 2-OG as amino group acceptor for their characterization of AtDAT1, our results revealed a higher V_max_ with pyruvate as substrate as compared to 2-OG. Most interestingly, the different enzymatic activities with pyruvate and 2-OG as amino group acceptors with ratios of 100:1 and more were in a comparable range as the D-Ala/D-Glu ratios found in plants after D-AA application (Gördes et al., 2011). Nevertheless, we could approximately substantiate the results from Funakoshi et al. (2008) when we used 2-OG as substrate in our enzymatic assays.

**Figure 9:**
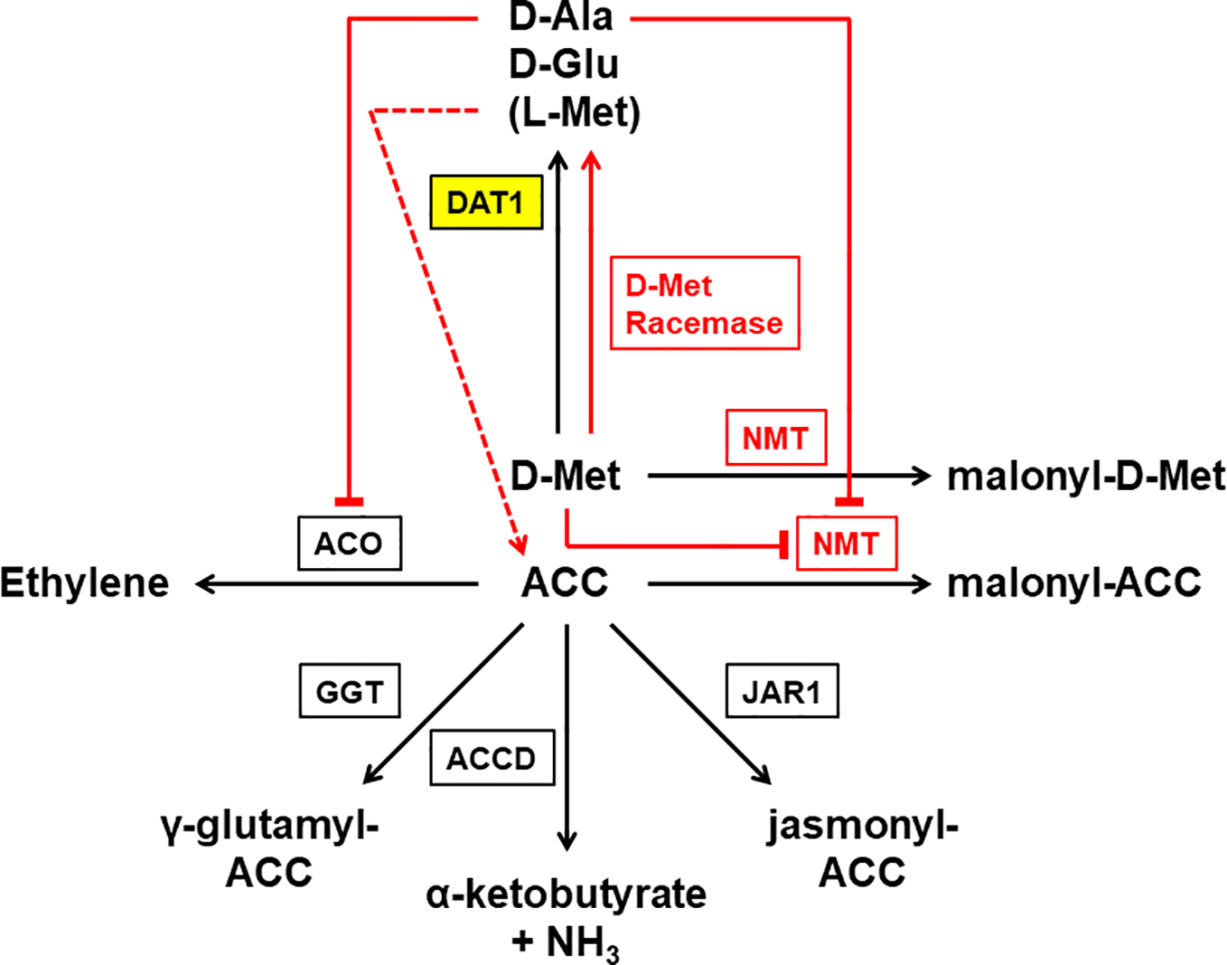
Working model of the different reactions leading to D-Met stimulated ethylene production in plants. This reaction scheme summarizes proven (black) and postulated (red) enzymes and reactions involved in the process of D-Met stimulated ethylene production in plants. As the central enzyme of this study DAT1 is highlighted in yellow. Externally applied D-Met is mainly transaminated by a D-amino acid transaminase (DAT1) to produce D-Ala and D-Glu. Additionally, L-Met is produced that mainly results from a second transamination step after transamination of D-Met. ACC is the precursor of the gaseous hormone ethylene and this reaction is catalyzed by the ACC oxidase (ACO). Alternative to transamination, D-Met is malonylated by a N-malonyl transferase (NMT), which also uses ACC as a substrate. The malonylation of D-Met by NMT leads to the competitive repression of the reaction with ACC. The consequence of DAT1 loss of activity would be an increase of D-Met concentration, which would repress ACC malonylation and lead to increased ethylene production. Although malonylation is thought to be the major route to regulate cellular ACC concentration, there are three additional ways known: the glutamylation of ACC by the γ-glutamyl transpeptidase (GGT), the addition of jasmonic acid to ACC by jasmonic acid resistance 1 (JAR1), and the deamination of ACC by the ACC deaminase (ACCD). But also two other metabolites of D-Met may affect ACC and ethylene levels: D-Ala is able to inhibit ACO but would be missing in case of DAT1 loss. In contrast, L-Met is a precursor of ACC and may also be produced by direct racemization from D-Met. Higher concentrations of L-Met by such a racemization may also lead to an increase of ACC levels even when DAT1 activity is decreased.

A major question in our studies addressed the role of AtDAT1 in D-AA stimulated ethylene production. As it is demonstrated here, this phenomenon is tightly connected to AtDAT1 (**Figure 9**). The loss of DAT1 leads to a significant increase of ethylene after D-Met application, resulting primarily in shortening of the hypocotyl and root in the *dat1* mutants and L*er* irrespective of the light regime. D-Met application also led to an increased production of malonyl-methionine, especially in the *dat1* mutants and L*er*, and the amount of malonyl-ACC developed reciprocally in all tested lines. The reciprocal accumulation of malonylated D-Met and ACC implies that the loss of *AtDAT* function or enzymatic activity results in over-accumulation of ACC that is then causing an increased ethylene production and eventually reduced seedling growth. However, this conclusion has to be reviewed critically, because even the *dat1* mutant seedlings did not show the full spectrum of the canonical triple response, as tightening of the apical hook or thickening of the hypocotyl was only partially observed. Furthermore, no differences in levels of malonylated ACC were detected between D-Met treated Col-0 and *dat1* mutants. However, it must be noted that in all previous studies the production of ethylene in response to malonylation of ACC and D-AAs were measured during overnight feeding experiments. The physiological growth responses and the contents of D-Met, ACC, and their malonylated derivatives in the plants over a longer period of the treatment were shown here for the first time.

The increased production of malonyl-methionine and ethylene without a decreased malonyl-ACC production in the *dat1* mutants in comparison to the control raises the question whether the original working model of D-Met stimulated ethylene production needs additional factors or metabolic processes. One explanation may be that malonylation is not the only way to regulate the ACC level in plants (**Figure 9**). There is also the possibility that ACC is conjugated with glutathione to γ-glutamyl-ACC (GACC) and with jasmonic acid to JA-ACC to control the ACC homeostasis. Additionally, plants can irreversibly degrade ACC to α-ketobutyrate by an ACC deaminase [for reviews about ACC content regulation, see Van de Poel and Van Der Straeten (2014); Le Deunff and Lecourt (2016), and Vanderstraeten and Van Der Straeten (2017)]. To date, neither the contribution of each of these ACC catabolic pathways nor their interplay for the control of ACC homeostasis have been studied, yet. It remains to be investigated whether D-Met, its malonylated form, or the loss of this way to degrade D-Met have an impact also on the alternative ACC degradation pathways.

Another explanation would be given by a racemization of D-Met, which has been proposed before but never been proven (Vranova et al., 2012; Gördes et al., 2013). Our results imply that the majority of the increase of L-Met in D-Met fed Col-0 plants arises from the reamination of 4-methylthio-2-oxobutanoate after removal of the amino group from D-Met by AtDAT1. However, direct racemization of D-Met cannot be excluded. If such a direct racemization really exists, additional L-Met would be produced irrespective of AtDAT1 activity and would be partially converted to ACC in the Yang cycle (**Figure 9**). This additional ACC would contribute to the increased ethylene contents in D-Met treated *dat1* mutants, because ACC malonylation is inhibited competitively by higher D-Met levels.

A third explanation may be given by the effect of the AtDAT1 enzymatic products on the activity of other enzymes. Here we demonstrated that the loss of this enzyme leaves *dat1* mutants without the ability to produce D-Ala, D-Glu and additional L-Met in response to D-Met. Most interestingly, it was shown previously that D-Ala inhibits the ACC oxidase (ACO) (Gibson et al., 1998; Brunhuber et al., 2000; Charng et al., 2001; Thrower et al., 2006). This means that plants with functional DAT1 would malonylate D-Met instead of ACC but the produced D-Ala would partially inhibit the ACO and as one consequence the additional ACC would just be partially converted to ethylene. In the *dat1* mutants this inhibiting effect of D-Ala would be lost and may explain the ethylene increase in these lines in comparison to the corresponding wild type. The same lack of D-Ala accumulation may also contribute to the higher content of malonyl-methionine in the *dat1* mutants and to the comparable amount of malonylated ACC in all tested lines: D-Ala also partially inhibits the putative malonyl transferase (Kionka and Amrhein, 1984; Liu et al., 1985; Chick and Leung, 1997). The malonylation of D-Met would then be limited by D-Ala in Col-0 but not in the *dat1* mutant lines and L*er*. If D-Met is the preferred substrate of the malonyl transferase, the lack of significant differences between the tested lines in their levels of malonylated ACC would not be surprising. To confirm the assumptions that D-Ala influences the D-Met stimulated ethylene production by inhibiting the ACC oxidase, the ACC malonyl transferase or both enzymes, further physiological experiments with D-Ala and structural analogs like D-cycloserine are required. However, the final answer to this question is awaited by the identification of the ACC malonyl transferase and the results of the enzyme´s biochemical and physiological characterization.

Undoubtedly, AtDAT1 affects D-Met stimulated ethylene production and seems to have quite specific effects in this regard. However, as the working model in **Figure 9** implies, the relationship between D-Met and ethylene may be more complex than just the competition for the N-malonyl transferase (NMT). As mentioned above, D-Met may affect the levels of ACC and all its derivatives. It has been shown before that ACC itself acts as a signaling molecule and the same is also discussed for its derivatives (for reviews, see Van de Poel and Van Der Straeten, 2014; Vanderstraeten and Van Der Straeten, 2017; Nascimento et al., 2018). D-Met accumulation leads to an increase of ethylene concentrations, but possibly other compounds like ACC and its derivatives may also contribute to the observed physiological responses of *dat1* affected plants. This would explain why the *dat1* mutants do not show the full spectrum of triple response after treatment in the presence of D-Met. Detailed flux measurements of ACC and its derivatives after D-Met application as well as studies of *dat1* alleles in the background of ethylene synthesis and receptor mutants may shed more light on this aspect.

In this regard also the intracellular localization of these biochemical processes is of interest. The localization of AtDAT1 implies that the transamination takes place in the chloroplast, whereas the ACC oxidation is postulated to happen either in the cytosol or the plasma membrane (Houben and Van De Poel, 2019). The separation of these processes raises the question how D-Met affects malonylation of ACC if it is also located in the cytosol. The most apparent hypothesis would be that chloroplasts have a certain capacity to take up D-Met. Flooding of the chloroplasts with this compound could therefore lead to inhibitory processes in the cytosol. This would be supported by the findings in this study that AtDAT1 is the major enzyme to degrade D-Met but needs further confirmation.

Another remaining question is the source of D-Met in nature, because it was not reported in plants until now. In contrast, it was demonstrated previously that D-Met is released by bacterial biofilms into the environment (Kolodkin-Gal et al., 2010; Vlamakis et al., 2013) and that different rhizosphere colonizing bacterial species are able to utilize D-Met as sole carbon and nitrogen source (Radkov et al., 2016). Biofilm formation on root surfaces as a bacterial pathogen protection strategy was reported before (Vlamakis et al. (2013). It is remarkable that D-Met is released by different bacterial species into their growth media to concentrations up to 300–500 µM (Lam et al., 2009), which would match the most effective D-Met concentrations in our study. Possibly, AtDAT1 is part of bacterial biofilm recognition and therefore may be involved in plant–bacterial interaction.

This possibility would also offer an explanation why *AtDAT1* is dispensable in particular *Arabidopsis* accessions such as L*er* and M7323S. An explanation for the dispensability of the D-Met catabolic function of AtDAT1 would be that in a habitat with only minor D-Met releasing bacteria in the rhizosphere, a recognition system for this compound would be also dispensable for the plant. However, this needs to be tested. The viability of *Arabidopsis dat1* mutants and accessions without functional AtDAT1 also argues against the crucial function of this enzyme in folate biosynthesis. This was implied by the observation that the only known enzyme able to synthesize p-amino benzoic acid (pABA), the substructure of folates, is AtDAT1 (Basset et al., 2004; Hanson and Gregory III, 2011). Consequently, the loss of this enzyme would lead to the inability to produce essential folate which would reduce the plant viability dramatically. Interestingly, pABA is also involved in the regulation of root gravitropism (Nziengui et al., 2018). This implies a modulatory role of AtDAT in differential root growth including gravitropism, which can be tested in future by the analysis of our *dat1* mutants and accessions without or reduced AtDAT1 activity. Interestingly, DAT1 encoding genes seem to be found in all sequenced plant genomes (for a selection, see **Figure S5**), and ethylene production in other plant species than *Arabidopsis* is also induced by other D-AAs like D-Leu, D-Thr, D-Val, or D-Phe (Satoh and Esashi, 1980; Satoh and Esashi, 1982; Liu et al., 1983). In this regard, it would be interesting if also other D-AAs than D-Met cause growth defects and ethylene production in *dat1* mutants. Furthermore, it should be tested if the DAT1 enzymes from different species have altered substrate specificities and therefore contribute to the adaptation of plants to changing microbial environments.

## Data Availability Statement

All datasets generated for this study are included in the article/**Supplementary Material**.

## Author Contributions

ÜK, JS, and CH designed the study. JS and CH conducted most of the experiments and contributed equally to the study. V-AL and SH conducted another part of the experiments. CH and MS analyzed the biochemical data and ÜK wrote the manuscript.

## Funding

JS was supported by the Deutscher Akademischer Austauschdienst (DAAD 91567028). We acknowledge support by Open Access Publishing Fund of University of Tübingen.

## Conflict of Interest

The authors declare that the research was conducted in the absence of any commercial or financial relationships that could be construed as a potential conflict of interest.
