# Supplementary figures and images for: AtDAT1 Is a Key Enzyme of D-Amino Acid Stimulated Ethylene Production in Arabidopsis thaliana
Source: Front Plant Sci. 2019 Dec 12;10:1609. doi: 10.3389/fpls.2019.01609 (PMC6921899; doi:10.3389/fpls.2019.01609)

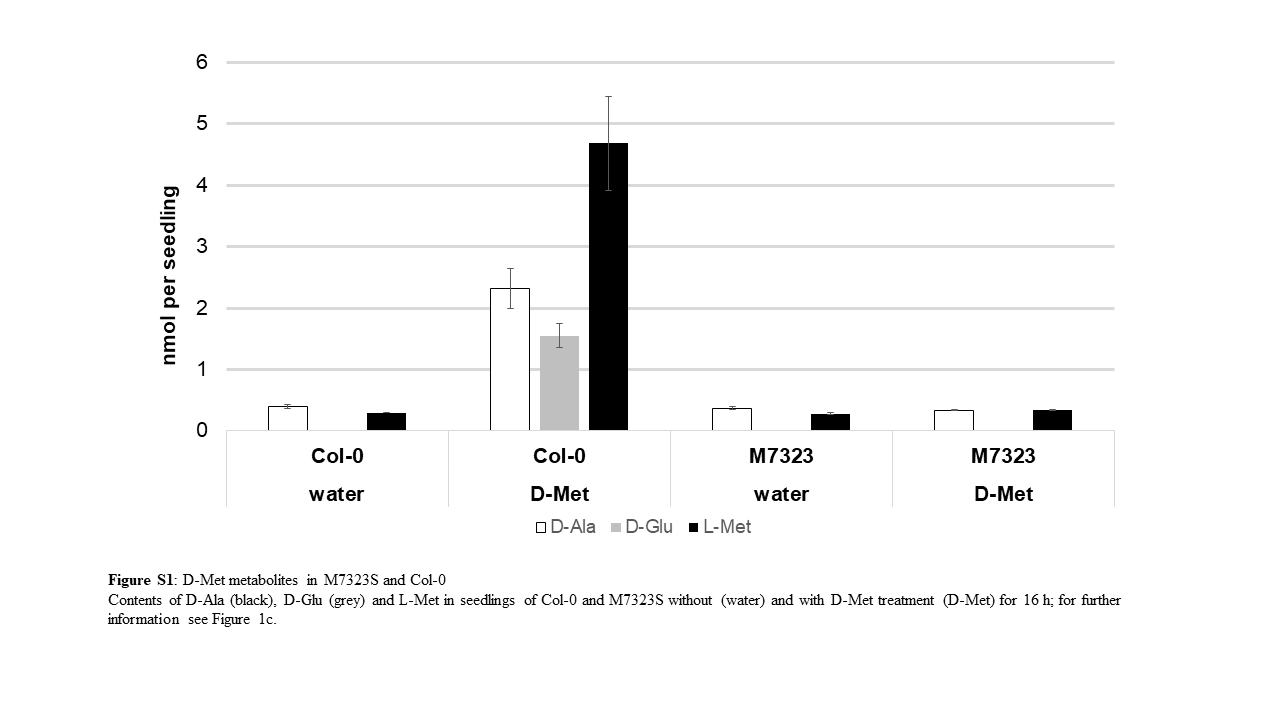

Supplement: Supplementary file 2 [file Image_1.tif]

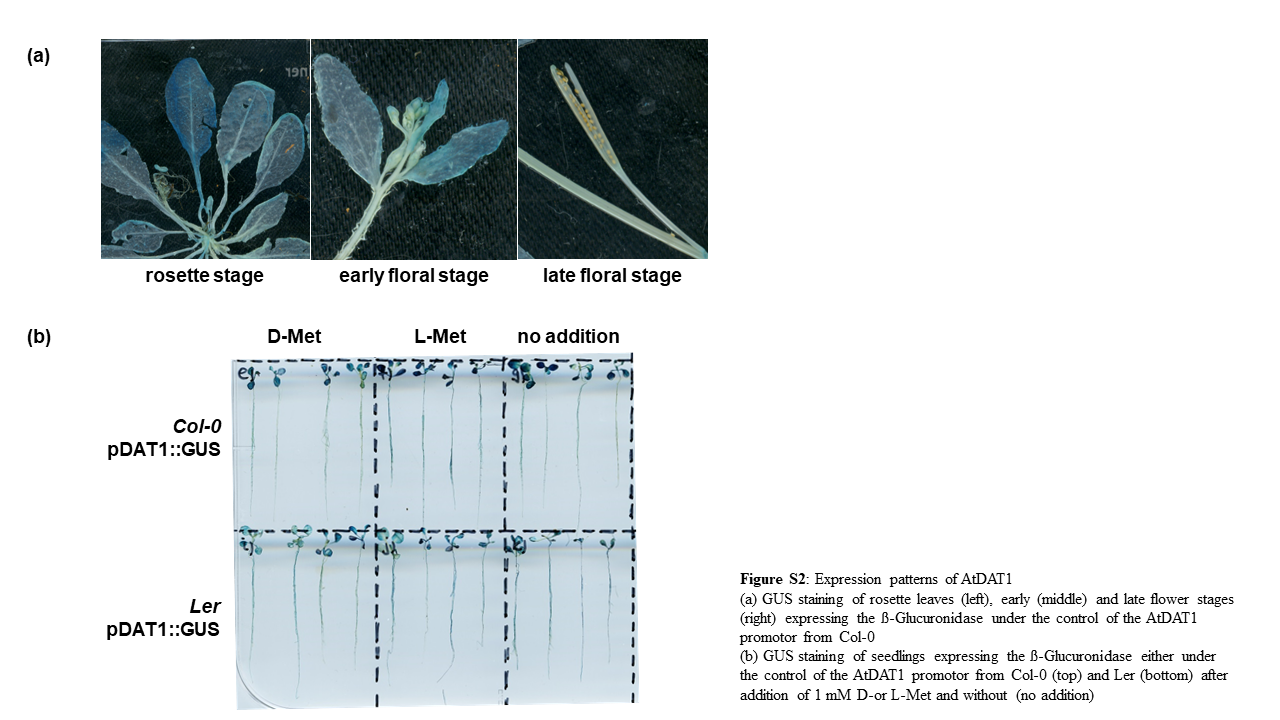

Supplement: Supplementary file 3 [file Image_2.tif]

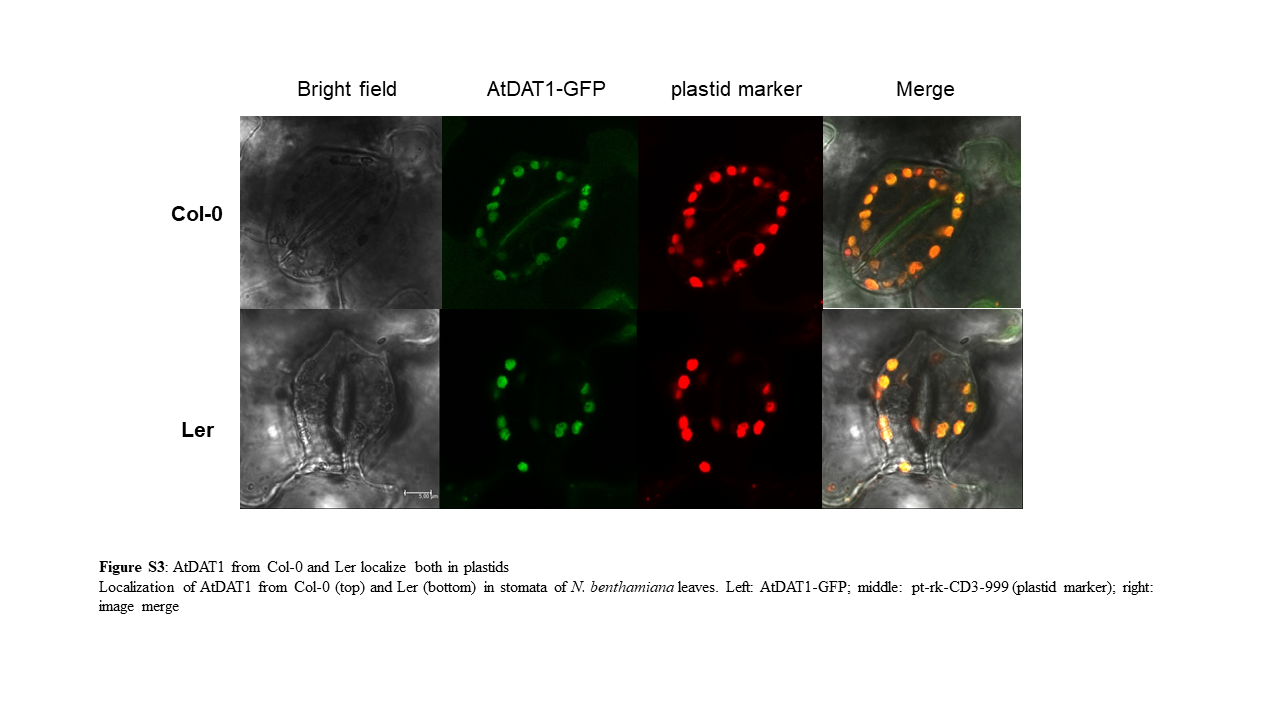

Supplement: Supplementary file 4 [file Image_3.tif]

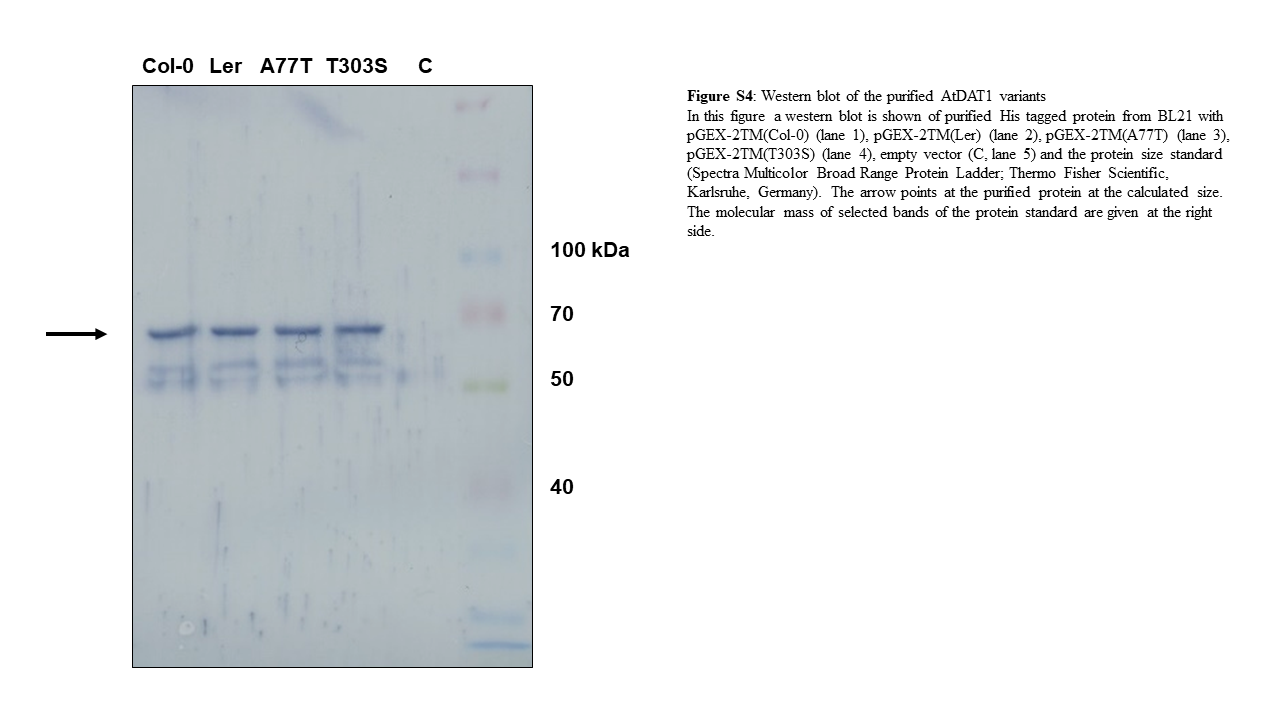

Supplement: Supplementary file 5 [file Image_4.tif]

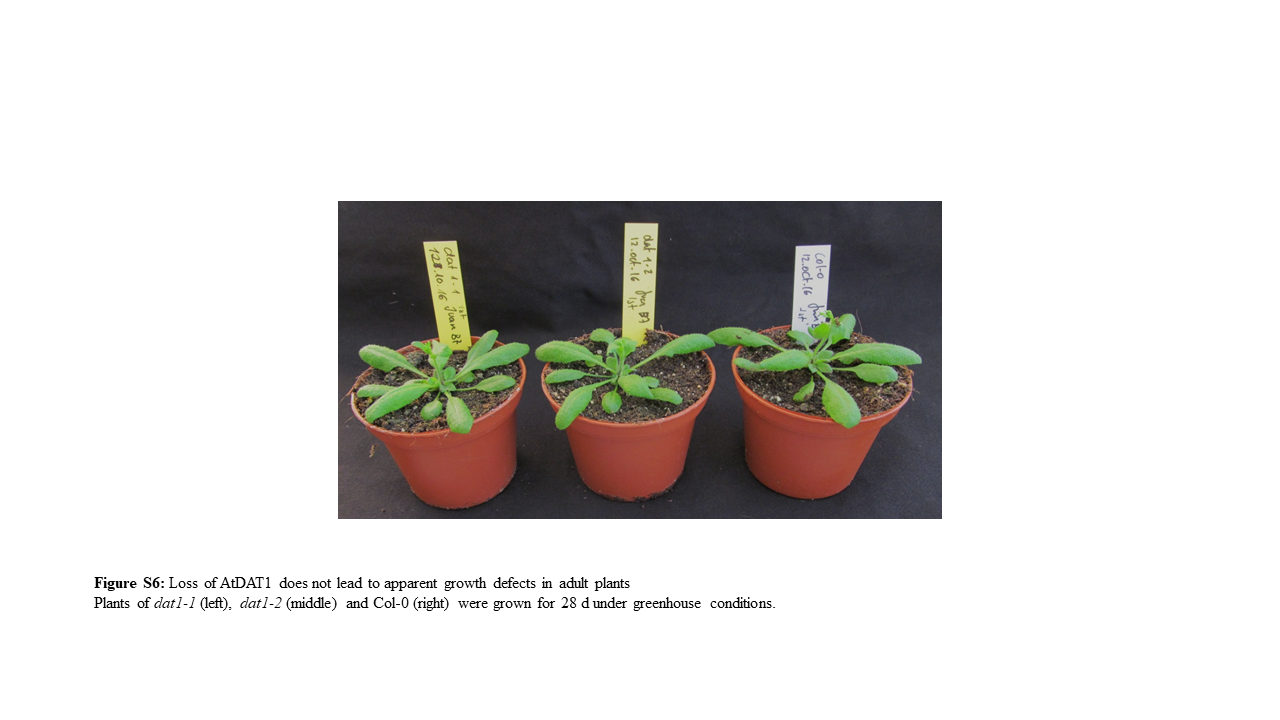

Supplement: Supplementary file 7 [file Image_6.tif]

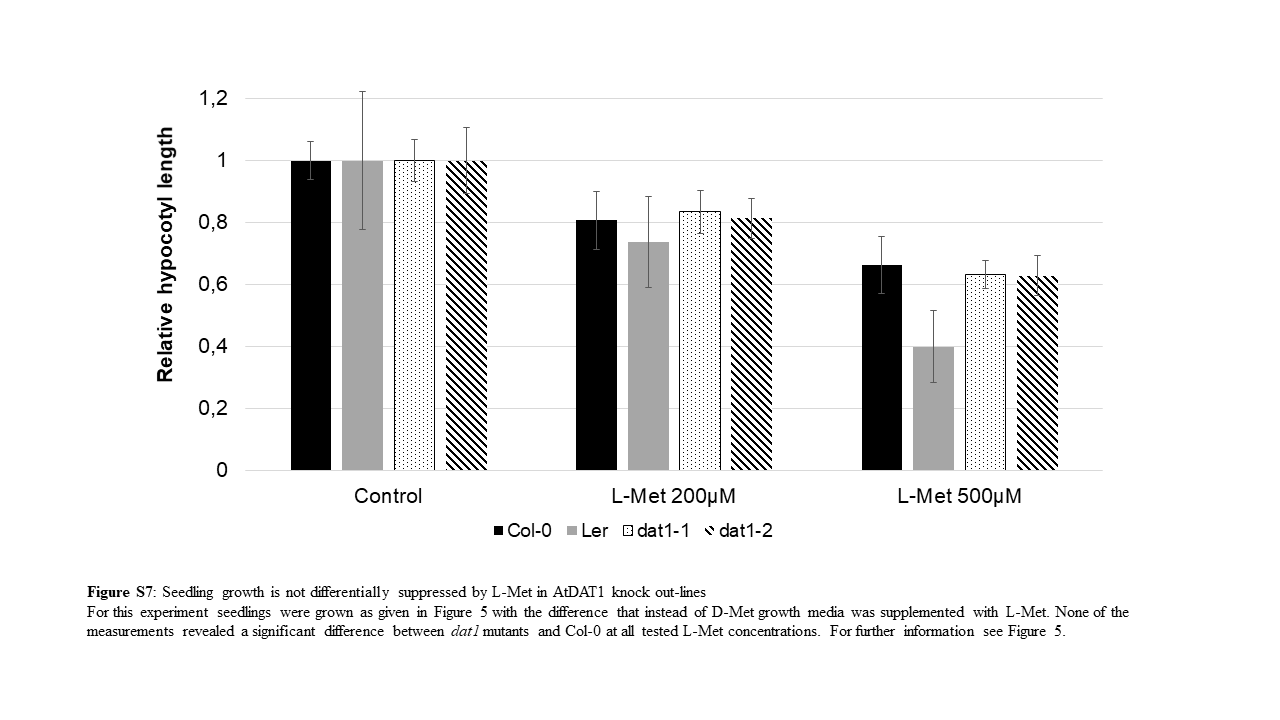

Supplement: Supplementary file 8 [file Image_7.tif]
