# Supplementary material for: AtDAT1 Is a Key Enzyme of D-Amino Acid Stimulated Ethylene Production in Arabidopsis thaliana
Source: Front Plant Sci. 2019 Dec 12;10:1609. doi: 10.3389/fpls.2019.01609 (PMC6921899; doi:10.3389/fpls.2019.01609)
Supplement: Supplementary file 6 [file Image_5.pdf]

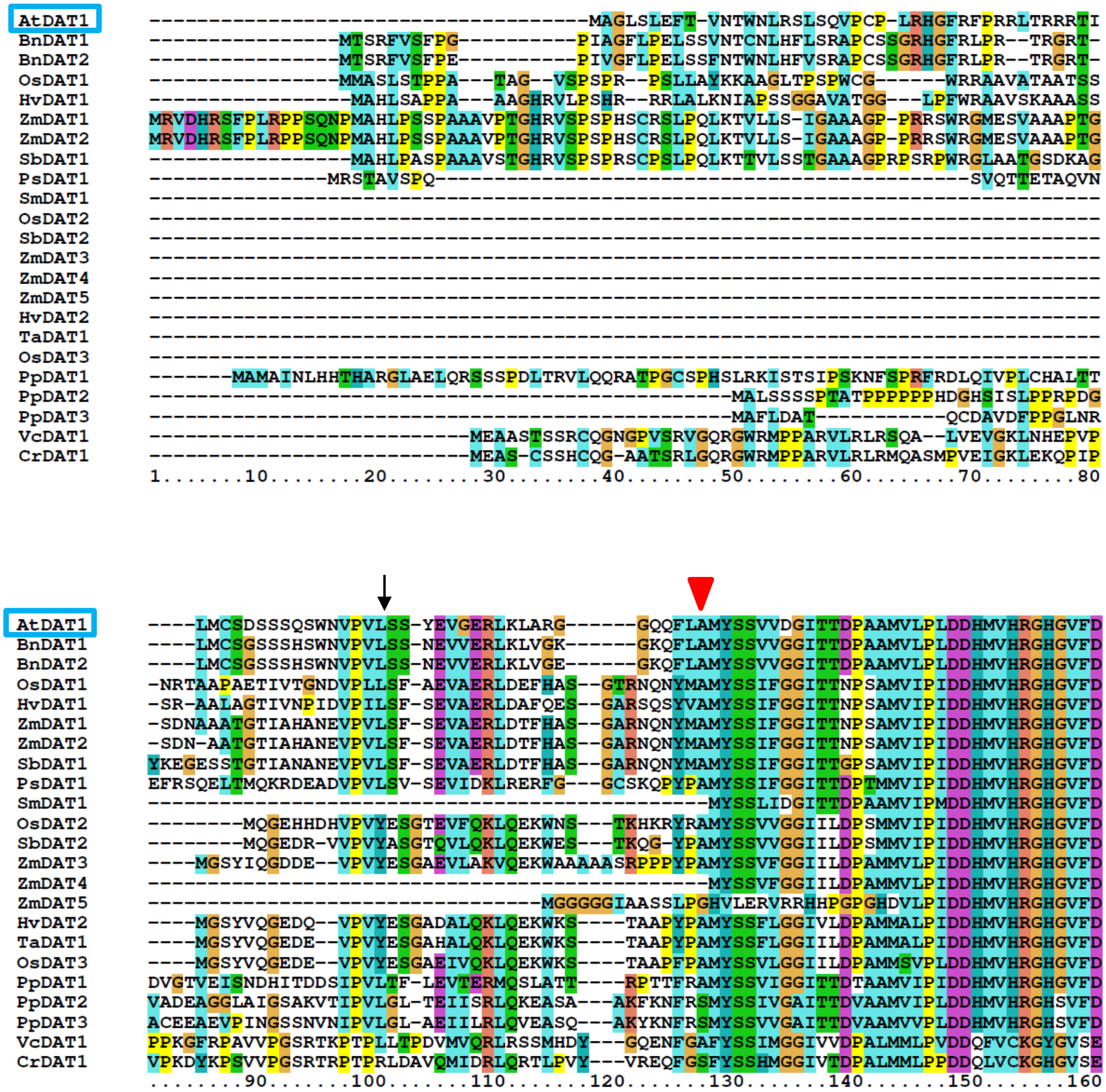
AtDAT1 -------------------------------------MAGLSLEFT-VNTWNLRSLSQVPCP-LRHGFRFPRRLTRRRTI
BnDAT1 ----------------MTSRFVSFPG----------PIAGFLPELSSVNTCNLHFLSRAPCSSGRHGFRLPR--TRGRT-
BnDAT2 ----------------MTSRFVSFPE----------PIVGFLPELSSFNTWNLHFVSRAPCSSGRHGFRLPR--TRGRT-
OsDAT1 ----------------MMASLSTPPA---TAG--VSPSPR--PSLLAYKKAAGLTPSPWCG------WRRAAVATAATSS
HvDAT1 -----------------MAHLSAPPA---AAGHRVLPSHR--RRLALKNIAPSSGGAVATGG---LPFWRAAVSKAAASS
ZmDAT1 MRVDHRSFPLRPPSQNPMAHLPSSPAAAVPTGHRVSPSPHSCRSLPQLKTVLLS-IGAAAGP-PRRSWRGMESVAAAPTG
ZmDAT2 MRVDHRSFPLRPPSQNPMAHLPSSPAAAVPTGHRVSPSPHSCRSLPQLKTVLLS-IGAAAGP-PRRSWRGMESVAAAPTG
SbDAT1 -----------------MAHLPASPAAAVSTGHRVSPSPRSCPSLPQLKTTVLSSTGAAAGPRPSRPWRGLAATGSDKAG
PsDAT1 ---------------MRSTAVSPQ---SVQTTETAQVN
SmDAT1 --
OsDAT2 --
SbDAT2 --
ZmDAT3 --
ZmDAT4 --
ZmDAT5 --
HvDAT2 --
TaDAT1 --
OsDAT3 --
PpDAT1 -------MAMAINLHHTHARGLAELQRSSSPDLTRVLQQRATPGCSPHSLRKISTSIPSKNFSPRFRDLQIVPLCHALTT
PpDAT2 ---MALSSSSPTATPPPPPPHDGHSISLPPRPDG
PpDAT3 ---MAFLDAT-----------QCDAVDFPPGLNR
VcDAT1 ---------------------------MEAASTSSRCQGNGPVSRVGQRGWRMPPARVLRLRSQA--LVEVGKLNHEPVP
CrDAT1 ---------------------------MEAS-CSSHCQG-AATSRLGQRGWRMPPARVLRLRMQASMPVEIGKLEKQPIP
1.......10........20........30........40........50........60........70........80
AtDAT1 ----LMCSDSSSQSWNVPVLSS-YEVGERLKLARG------GQQFLAMYSSVVDGITTDPAAMVLPLDDHMVHRGHGVFD
BnDAT1 ----LMCSGSSSHSWNVPVLSS-NEVVERLKLVGK------GKQFLAMYSSVVGGITTDPAAMVLPLDDHMVHRGHGVFD
BnDAT2 ----LMCSGSSSHSWNVPVLSS-NEVVERLKLVGE------GKQFLAMYSSVVGGITTDPAAMVLPLDDHMVHRGHGVFD
OsDAT1 -NRTAAPAETIVTGNDVPLLSF-AEVAERLDEFHAS--GTRNQNYMAMYSSIFGGITTNPSAMVIPIDDHMVHRGHGVFD
HvDAT1 -SR-AALAGTIVNPIDVPILSF-SEVAERLDAFQES--GARSQSYVAMYSSIFGGITTNPAAMVIPIDDHMVHRGHGVFD
ZmDAT1 -SDNAAATGTIAHANEVPVLSF-SEVAERLDTFHAS--GARNQNYMAMYSSIFGGITTNPSAMVIPIDDHMVHRGHGVFD
ZmDAT2 -SDN-AATGTIAHANEVPVLSF-SEVAERLDTFHAS--GARNQNYMAMYSSIFGGITTNPSAMVIPIDDHMVHRGHGVFD
SbDAT1 YKEGESSTGTIANANEVPVLSF-SEVAERLDTFHAS--GARNQNYMAMYSSIFGGITTGPSAMVIPIDDHMVHRGHGVFD
PsDAT1 EFRSQELTMQKRDEADVPVLSV-SEVIDKLRERFG---GCSKQPYPAMYSSIFGGITTDPTMMVIPIDDHMVHRGHGVFD
SmDAT1 ---MYSSLIDGITTDPAAMVIPMDDHMVHRGHGVFD
OsDAT2 --------MQGEHHDHVPVYESGTEVFQKLQEKWNS---TKHKRYRAMYSSVVGGIILDPSMMVIPIDDHMVHRGHGVFD
SbDAT2 --------MQGEDR-VVPVYASGTQVLQKLQEKWES---TKQG-YPAMYSSVVGGIILDPSMMVIPIDDHMVHRGHGVFD
ZmDAT3 ----MGSYIQGDDE--VPVYESGAEVLAKVQEKWAAAAASRPPPYPAMYSSVFGGIILDPAMMVLPIDDHMVHRGHGVFD
ZmDAT4 ---MYSSVFGGIILDPAMMVLPIDDHMVHRGHGVFD
ZmDAT5 ---------------------------------MGGGGGIAASSLPGHVLERVRRHHPGPGHDVLPIDDHMVHRGHGVFD
HvDAT2 ----MGSYVQGEDQ--VPVYESGADALQKLQEKWKS----TAAPYPAMYSSFLGGIVLDPAMMALPIDDHMVHRGHGVFD
TaDAT1 ----MGSYVQGEDE--VPVYESGAHALQKLQEKWKS----TAAPYPAMYSSFLGGIILDPAMMALPIDDHMVHRGHGVFD
OsDAT3 ----MGSYVQGEDE--VPVYESGAEIVQKLQEKWKS----TAAPFPAMYSSVLGGIILDPAMMSVPLDDHMVHRGHGVFD
PpDAT1 DVGTVEISNDHITDDSIPVLTF-LEVTERMQSLATT----RPTTFRAMYSSVIGGITTDTAAMVIPIDDHMVHRGHGVFD
PpDAT2 VADEAGGLAIGSAKVTIPVLGL-TEIISRLQKEASA---AKFKNFRSMYSSIVGAITTDVAAMVIPLDDHMVHRGHSVFD
PpDAT3 ACEEAEVPINGSSNVNIPVLGL-AEIILRLQVEASQ---AKYKNFRSMYSSVVGAITTDVAAMVVPLDDHMVHRGHSVFD
VcDAT1 PPKGFRPAVVPGSRTKPTPLLTPDVMVQRLRSSMHDY---GQENFGAFYSSIMGGIVVDPALMMLPVDDQFVCKGYGVSE
CrDAT1 VPKDYKPSVVPGSRTRPTPRLDAVQMIDRLQRTLPVY---VREQFGSFYSSHMGGIVTDPALMMLPPDDQLVCKGHGVSE
........90.......100.......110.......120.......130.......140.......150.......160

```
AtDAT1     TALIINGYLYELDQHLDRILRSASMAKIPL-PFDRETIKRILIQTVSVSGCRDGSLRYWLSAGPGDFLLSPSQCLKPTLY
BnDAT1     TAMVIKGYLYELDQHLDRILRSASMAKIPL-PFDRETIRSILIQTVSVSGCRYGSLRYWLSAGPGDFSLSPSQCPKPSLY
BnDAT2     TAMIVNGHLYELDQHLDRIIRSASMAKIPL-PFDRETIRRILIQTVSVSGCRYGSLRYWLSAGPGDFSLSPSQCPKPSLY
OsDAT1     TAAIMNGHLYELEQHLDRFLKSASMAKITL-PFDRSTIRSILIQTVSASKCTQGSLRYWLSVGPGDFQLSSAGCANSALY
HvDAT1     TAAIMDGHLYELEQHIDRFLNSAQMAKIPL-PFDRSKIRSVLIQTVCASKCSQGSLRYWLSVGPGDFQLSSSGCRNPALY
ZmDAT1     TAAIMDGHLYELEQHLDRFLKSASMAKIPL-PFNRSTIRSILIQTVSASNCTQGSLRYWLSAGPGDFQLSSSGCTNPALY
ZmDAT2     TAAIMDGHLYELEQHLDRFLKSASMAKIPL-PFNRSTIRSILIQTVSASNCTQGSLRYWLSAGPGDFQLSSSGCTNPALY
SbDAT1     TAAIMDGHLYELEQHLDRFLRSALMAKIPL-PFDRSTIRSILIQTVSASNCTQGSLRYWLSVGPGDFQLSSSGCANPALY
PsDAT1     TAMIIDGYLYELDSHLDRFLRSASKAKVIP-PFDRSSVRSILLQTVAASQCRKGSLRYWLSAGPGDFLLSPAGCPNSAFY
SmDAT1     TATIADGYLYELDAHLDRFHKSAAAAKIQP-PFDRATMREILIQTVASSGCKLGSLRYWLSAGPGGFGLSSSECTMSTLY
OsDAT2     TAMLSDGYLYELDSHLDRLLLSASKAKISS-PFSRETLRAILVQMTAASKCRNGSIKYWLSAGPGDFLLSPKGCTAPAFY
SbDAT2     TATISDGYLYELDSHLDRLLVSASKAKIDP-PFPRETLRNILLQMTAASGCKNGSIKYWLSAGPGDFLVSPKGCTGSAFY
ZmDAT3     TAMILDGALYELDAHLDRFLRSAAAARVGTAPFPREALRRILIQMTAASGCRMGSIRYWLSSGPGDFLLSSRGCPSPAFY
ZmDAT4     TAMILDGALYELDAHLDRFLRSAAAARVGTAPFPREALRRILIQMTAASGCRMGSIRYWLSSGPGDFLLSSRGCPSPAFY
ZmDAT5     TAMILDGALYELDAHLDRFLRSAAAARVGTAPFPREALRRILIQMTAASGCRMGSIRYWLSSGPGDFLLSSRGCPSPAFY
HvDAT2     TAMLLDGHLYELDAHLDRFLRSAAQAKVGT-PFPRDTLRSILVQMTAASGCRKGSIRYWLSSGPGDFLLSSSGCPGPAFY
TaDAT1     TAMLLDGHLYELDAHLDRFLRSAAQAKVGT-PFPRDTLRSILVQMTAASGCRKGSIRYWLSSGPGDFLLSSSGCPGPAFY
OsDAT3     TAMILDGHLYELDPHLDRFLRSAAKARIGT-PFPRDTLRSILVQMTAASNCRRGSIRYWLSAGGGDFLLSSAGCAGPAFY
PpDAT1     TAIIYNGYLYELDDHLDRILRSAEKAKIQS-PFDRATLRDILVQTVAASGCRRGALRYWLSAGLGGFALSSKECFKSTFY
PpDAT2     TSILVNGYLYELDAHLDRFLSSATKAKITP-PFDRATIREILLQTVSAGKCQHGILRFWMSVGRGNFELSAKNCLESSLF
PpDAT3     TTTLVNGNLYELDTHLDRFLDSAAKAKILP-PFNRAMIREILMQTVAAGSCKDGTLRFWLSAGRGNFELSTKNC-EASLY
VcDAT1     TVVLRDGHLYMLDEHIARLTAACAQVGLSL-PFSVPAVKRIVLDTAAASGKLNGQLRFWVTPGRGGFSPVELGGSEPALY
CrDAT1     VVVLREGHIYLLDRHIQRLKESCEQVGIAL-PFEEASLKRILLDVAAASGRVNGVVRFWATPGRGGFSTVETGGAEPAFY
           .......170.......180.......190.......200.......210.......220.......230.......240
```

```
AtDAT1     AIVIKTNFAINP-------IGVKVVTSSIPIKPPEFATVKSVNYLPNVLSQMEAEAKGAYAGIWVCK--DGFIAEGPNMN
BnDAT1     AIVYQKDFTVDR-------RGVKVVTSSIPIKPPEFATVKSVNYLPNALSQMEAEAKGAYAGIWVDN--DGFIAEGPNMN
BnDAT2     AIVYQKDFTVDR-------RGVRVVTSSIPIKPPEFATVKSVNYLPNALSQMEAEAKGAYAGIWVDN--DGFIAEGPNMN
OsDAT1     AIVIESPSLPVP-------AGCKVITSSIPIKSQQFAVMKSVNYLPNALTKVEGEENGGFTGIWLDD--EGFVAEGSNMN
HvDAT1     AVVIESPSLPEP-------SGCKVITSSIPVKSPQFAVMKNVNYLPNALTKVEGEENGGFTGIWLDD--EGFVAEGSNMN
ZmDAT1     AVVIESPSLQVP-------SCCRVVTSSIPIKSPQFAVMKSVNYLPNALTKVEGEENGAFTGIWLDD--EGFVAEGSNMN
ZmDAT2     AVVIESPSLQVP-------SCCRVVTSSIPIKSPQFAVMKSVNYLPNALTKVEGEENGAFTGIWLDD--EGFVAEGSNMN
SbDAT1     AVVIESPSLQVP-------SCCKVVTSSIPIKSPQFAVMKSVNYLPNALTKVEGEENGAFSGIWLDD--EGFVAEGSNMN
PsDAT1     AIVIEDDYSPSS-------QGVTVITSSTPMKSPEMATMKNVNYLPNVLSKMEAEEKGAFAAIWIDD--QDYIAEGPNVN
SmDAT1     AVVLSLPAIPDPD------MGVTVITASTPMKHPQFATMKNVNYLPNALSKLEAESQGAFAAIWLDD--EGFVAEGPNVN
OsDAT2     AVVIASAAAAAAGGHPRLREGVRAITSTVPMKDPFFAAMKSVNYLANALAMAEAEERGAYASVWVDG--DGGVAEGPMMN
SbDAT2     AVV-----AAAAGGYRHKDGGVKAITATVPMKHPFFAGIKSVNYLPNALAMAEAEERGAFASVWVDE--DGYVAEGPTMN
ZmDAT3     GVVIASEYEQCG----VDGTGVRAVTATVPMKPPQFATVKNVNYLPNVLSIMDAEDRGAFASVWVDD--QGYVAEGPMVN
ZmDAT4     GVVIASEYEQCG----VDGTGVRAVTATVPMKPPQFATVKNVNYLPNVLSIMDAEDRGAFASVWVDD--QGYVAEGPMVN
ZmDAT5     GVVIASEYEQCG----VDGTGVRAVTATVPMKPPQFATVKNVNYLPNVLSIMDAEDRGAFASVWVDD--QGYVAEGPMVN
HvDAT2     AVVIPSDYAQC-------RDGVRAVTTSVPMKPPLFATMKNVNYLPNVLSIMDAEERGAFASVWVDE--QGYVAEGPMVN
TaDAT1     AVAIPSDYAQC-------RDGVRAVTTSVPMKPPLFATMKNVNYLPNVLSIMDAEERGAFASVWVDE--QGYVAEGPMVN
OsDAT3     AVVIPTDYSQC-------RHGVRAVTTSVPMKPPLFATMKNVNYLPNVLSIMDAEDRGAFASVWVDG--EGNVAEGPMVN
PpDAT1     AIVTDNTYQGPE--------GVKVMTSTIPIKPPFFATVKSVNYLPNVLAQLEAEEKGLYAGIWLDN--EGFIAEGPNMN
PpDAT2     ACLVEEN---FIDDE--PLDGLKVITSTVPIKHKDFATIKSTNYLLNALVVMEAEEKGANVGIWLDE--DGNIAEGQNMN
PpDAT3     ACLLERSPIQNISDE--KVAPLKVVTSTVPIKPKMFATMKTTNYLPNALALREAEEKGAQAGIWLDE--EGNVAEGNNLN
VcDAT1     VICLGDTYEIDR------TESWDAILAEEPIQATAVSNVLGNQRLLTTVGQVEAHARDAHVALFTDA--EGFVQHCAGYT
CrDAT1     ALCLGDTYYMDR------MEAWPAMLVSQPVTPTLTSNVPGNQHLLTSVTQLIAGEEGMKATLFTDE--EGFVQHGSGFT
           .......250.......260.......270.......280.......290.......300.......310.......320
```

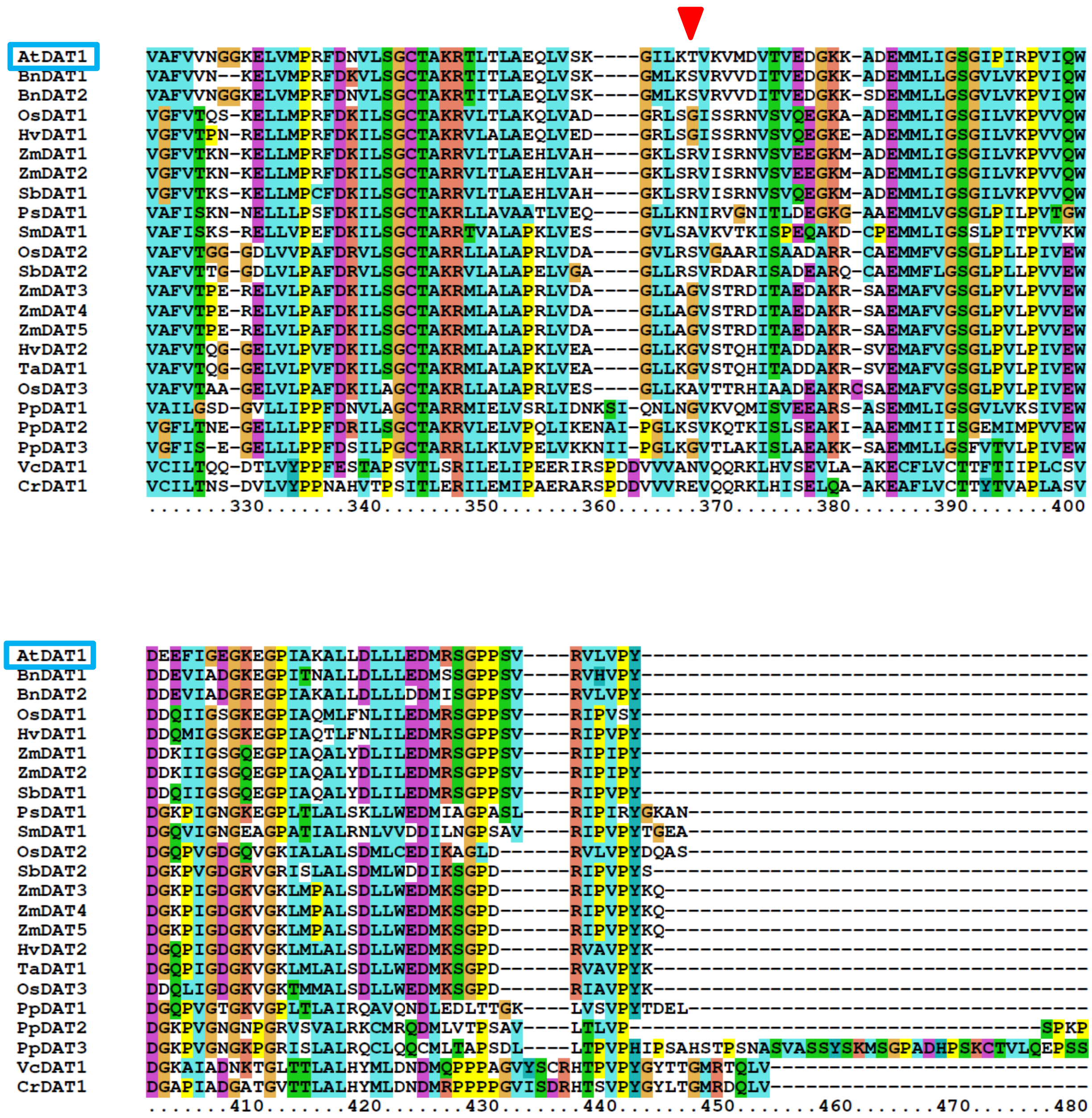

Figure S5: Alignment of DAT1 protein sequences from different plants and algae
Protein sequences were taken from the Phytozome 12 database (https://phytozome.jgi.doe.gov/pz/portal.html#) with AtDAT1 as search sequence. The alignment was constructed with ClustalX 2.1 (Larkin et al. 2007). AtDAT1 in the first line of the alignment is marked by a blue box. The chloroplastic transit peptide cleavage site (arrow) was determined according to the respective entry in the Plant Proteome Database (PPDB; http://ppdb.tc.cornell.edu/). Red triangles mark the sites of amino acid exchanges A77T and T303S between AtDAT1 from Col-0 and Ler. The blue star denotes the site of nonsense mutation in M7323S.
